# Integrative Transcriptomic and Metabolomic Analyses Reveal Molecular Processes Associated with Differential Embryo Dehydration in Maize

**DOI:** 10.3390/genes17101196

**Published:** 2026-09-27

**Authors:** Putong Wang, Tengjiao Jia, Long Zhang, Cheng Li, Jing Wang, Jiwei Yang, Zhanyi Zhang, Ziyu Qin, Jinxia Ma, Qizong Cai, Lifeng Wang, Huiyong Li

**Affiliations:** 1 Institute of Cereal Crops, Henan Academy of Agricultural Sciences, Zhengzhou 450002, China; wptwheat1994@126.com (P.W.); jiatengjiao1983@126.com (T.J.); swgczl@163.com (L.Z.); licheng_scau@126.com (C.L.); 18737617538@163.com (J.W.); yangjiwei2009@126.com (J.Y.); 13297099676@163.com (Z.Z.); qin123411@163.com (Z.Q.); 17839964218@163.com (J.M.); 18595852832@163.com (Q.C.); 2 State Key Laboratory of Maize Bio-Breeding, Zhengzhou 450002, China

**Keywords:** *Zea mays*, kernel moisture content, embryo dehydration, transcriptome, metabolomics, ABA signaling

## Abstract

Background/Objectives: Kernel dehydration is an important agronomic trait influencing mechanical harvesting efficiency, post-harvest drying costs, and grain storage quality in maize (*Zea mays* L.). Although the genetic and molecular basis of whole-kernel dehydration has been increasingly investigated, tissue-specific physiological and molecular patterns, particularly those associated with the embryo, remain insufficiently characterized. In this study, four maize inbred lines with contrasting dehydration capacities were selected to characterize tissue-specific dehydration dynamics and associated molecular patterns. Methods: Moisture content and dehydration rates of kernels, endosperms, and embryos were monitored from 10 to 55 days after pollination (DAP), followed by correlation analyses with kernel- and ear-related agronomic traits. Transcriptome sequencing, weighted gene co-expression network analysis (WGCNA), and untargeted metabolomics were integrated to characterize molecular processes associated with contrasting embryo dehydration phenotypes. Results: Embryo dehydration closely paralleled whole-kernel dehydration, and embryo dehydration rate was strongly associated with kernel dehydration rate. Transcriptomic and WGCNA analyses revealed developmental- and genotype-dependent expression changes, particularly during late maturation, and identified dehydration-associated modules enriched in lipid metabolism, glycolysis, hormone signaling, and stress-responsive processes, including fatty acid metabolism, ABA- and ethylene-related signaling, and MAPK-related genes. FDR-controlled metabolomics further revealed genotype- and developmental-stage-dependent metabolite differences associated with organic acid metabolism, amino acid metabolism, transport-associated processes, and secondary metabolism. Pathway-level integration highlighted ABA- and ethylene-associated candidate processes associated with variation in embryo dehydration. Conclusions: Collectively, these findings provide an embryo-focused characterization of physiological and multi-omics variation associated with maize kernel dehydration and identify candidate molecular processes for further functional investigation. Further validation across broader germplasm panels, environmental conditions, and experimental systems will be required to determine the causal roles and potential breeding relevance of these candidate processes.

## 1. Introduction

Maize is one of the world’s most important cereal crops, providing essential resources for human consumption, animal feed, and industrial applications. With the increasing adoption of mechanized harvesting, reducing kernel moisture content (KMC) at harvest has become an important breeding objective because excessive kernel moisture increases susceptibility to mechanical damage, increases the need for artificial drying, and compromises post-harvest storage quality [1,2]. During maturation, maize kernels undergo a developmental transition from active water accumulation to progressive dehydration and the acquisition of desiccation tolerance. The balance between water loss and retention ultimately influences grain moisture status, harvest efficiency, and post-harvest storage performance [3,4]. Extensive natural variation in kernel dehydration rate (KDR) and KMC has been observed among diverse maize germplasm, indicating that dehydration-related traits are under genetic control and represent promising targets for molecular breeding [5,6,7,8]. In addition, embryo moisture status influences kernel maturation, storage longevity, and subsequent germination performance [9,10]. However, most previous studies have investigated dehydration at the whole-kernel level, whereas the tissue-specific contributions to kernel water loss, particularly those of the embryo, remain poorly understood.

The maize kernel comprises three anatomically and functionally distinct tissues: the pericarp, endosperm, and embryo. The endosperm serves as the primary storage tissue, accumulating starch, proteins, and other reserves required for seed development and subsequent germination, whereas the embryo constitutes the living component of the seed and plays central roles in seed viability, germination capacity, and longevity [11,12]. During late seed maturation, the embryo undergoes extensive physiological and metabolic reprogramming associated with the establishment of dormancy and acquisition of desiccation tolerance. These processes involve the coordinated regulation of late embryogenesis abundant (LEA) proteins, osmoprotective metabolites, antioxidant defense systems, and membrane stabilization mechanisms [13,14,15,16]. In maize, embryo dehydration is closely associated with developmental maturation and the establishment of desiccation tolerance, suggesting that embryo water loss is an important component of seed maturation [17]. Distinct patterns of water regulation between embryos and endosperms may arise from tissue-specific differences in oxygen availability, respiratory metabolism, lipid turnover, and stress-responsive pathways during kernel development [18,19,20]. Therefore, elucidating the physiological characteristics and molecular mechanisms underlying embryo dehydration is essential for understanding tissue-specific regulation of maize seed maturation and kernel water loss dynamics.

Genetic analyses based on quantitative trait locus (QTL) mapping and genome-wide association studies (GWAS) have revealed the complex genetic architecture underlying maize dehydration-related traits [21,22]. Multiple QTLs associated with KDR and KMC have been identified, including major-effect loci such as *qGwc1.1*, which was fine-mapped to a 2.05-Mb interval, and *qFkdr7b*, a novel locus associated with rapid dehydration [23,24]. Meta-QTL analyses have further integrated results from independent mapping populations to refine dehydration-associated genomic regions [5]. Moreover, GWAS and multi-locus association analyses have identified numerous stable single-nucleotide polymorphisms (SNPs) and quantitative trait nucleotides (QTNs) associated with KDR and KMC across diverse maize germplasm, providing valuable genetic resources for dissecting the molecular basis of kernel dehydration and for facilitating molecular breeding. Recent functional studies have begun to identify key molecular regulators of maize dehydration regulation [21,25]. The *qKDR1* locus was recently shown to encode a maize-specific 31-amino-acid micropeptide, microRPG1, which promotes kernel dehydration by modulating ethylene signaling components EIL1 and EIL3 during post-filling maturation [26]. In addition, genes involved in starch biosynthesis, LEA protein accumulation, and ABA-responsive SnRK2 signaling have been implicated in kernel maturation and dehydration-related processes [27,28,29,30]. Collectively, these studies have established an emerging genetic and molecular framework for understanding maize kernel dehydration.

In this study, we investigated the physiological and molecular basis of variation in embryo dehydration among maize inbred lines with contrasting dehydration capacities. Four representative inbred lines were selected from our previously established GWAS population for grain drying rate [31], and temporal changes in moisture content and dehydration rate were systematically characterized in whole kernels, endosperms, and embryos. Correlation analyses were subsequently conducted to examine relationships between tissue-specific dehydration dynamics and kernel- and ear-related agronomic traits. To investigate molecular processes associated with embryo dehydration variation, transcriptome sequencing was performed on embryos collected at 35, 45, and 55 days after pollination (DAP), followed by weighted gene co-expression network analysis (WGCNA), untargeted metabolomic profiling, and pathway-level integrated transcriptomic and metabolomic analyses. These approaches enabled the identification of molecular processes and candidate pathways associated with contrasting embryo dehydration phenotypes. Our results reveal close physiological associations between embryo and whole-kernel dehydration and identify developmental- and genotype-dependent transcriptional and metabolic patterns associated with lipid metabolism, glycolysis, MAPK signaling, and ABA- and ethylene-associated regulatory processes. These findings provide an embryo-focused perspective on maize kernel dehydration and identify candidate molecular processes for further functional investigation.

## 2. Materials and Methods

### 2.1. Plant Materials and Field Experiments

A panel comprising 309 maize inbred lines with contrasting kernel dehydration capacities was previously established based on grain moisture content and dehydration rate [31]. From this panel, four representative inbred lines were selected for detailed physiological, transcriptomic, and metabolomic analyses, including two fast-dehydrating (FDR) genotypes (HCL645 and PHG71) and two slow-dehydrating (SDR) genotypes (911 and A314). These genotypes exhibited significant differences in kernel dehydration performance during late grain filling and maturation.

Field experiments were conducted at the experimental station in Yuanyang, Henan Province, China (35.012° N, 113.704° E). All genotypes were grown under uniform field conditions using a randomized complete block design. Standard agronomic practices, including fertilization, irrigation, and pest management, were applied throughout the growing season. To minimize environmental variation caused by uncontrolled pollination, ears were bagged before silk emergence, and manual self-pollination was performed on the same day. The date of pollination was designated as 0 DAP. Plants were grown under natural field conditions until physiological maturity.

### 2.2. Phenotypic Evaluation and Tissue-Specific Dehydration Analysis

To characterize tissue-specific dehydration dynamics during kernel development, developing kernels were collected at 10, 20, 30, 35, 40, 45, 50, and 55 DAP. Three biological replicates were collected for each genotype and developmental stage, with each replicate derived from independent plants. Fresh kernels were immediately weighed, and embryo and endosperm tissues were manually separated for tissue-level physiological measurements. The fresh weight (FW) of whole kernels and the corresponding tissues was recorded separately. Samples were subsequently oven-dried at 105 °C for 30 min and further dried at 80 °C until a constant weight was achieved [32]. Dry weight (DW) was then determined.

Moisture content (MC) was calculated as follows:

MC (%) = [(FW − DW)/FW] × 100,(1)

The dehydration rate (DR) between two consecutive developmental stages was calculated as:

DR (%) = (MC*t*1 − MC*t*2)/(*t*2 − *t*1), (2)

where *t*1 and *t*2 represent two consecutive sampling dates.

The area under the dehydration dynamics curve (AUDDC) was calculated using the trapezoidal integration method to evaluate cumulative dehydration performance, as previously described [33]. Lower AUDDC values indicate higher dehydration efficiency.

At physiological maturity, mature ears were harvested for agronomic trait evaluation. Three representative ears per genotype were collected, and ear-related traits, including ear length (EL), ear diameter (ED), kernel number per row (KNR), kernel row number (KRN), and kernel weight per ear (KWE), were measured. Kernel-related traits, including 100-kernel weight (100 KW), kernel length (KL), kernel width (KW), and kernel thickness (KT), were also evaluated.

### 2.3. RNA Extraction, Library Construction, and Transcriptome Sequencing

For transcriptomic analysis, embryo tissues were collected from kernels at 35, 45, and 55 DAP. Embryos were manually separated from kernels, immediately frozen in liquid nitrogen, and stored at −80 °C until RNA extraction. Three independent biological replicates were prepared for each genotype and developmental stage combination. Total RNA was extracted using TRIzol reagent (Invitrogen, Carlsbad, CA, USA) according to the manufacturer’s instructions. RNA concentration and purity were determined using a NanoDrop spectrophotometer (Thermo Fisher Scientific, Waltham, MA, USA), while RNA integrity was assessed using an Agilent 2100 Bioanalyzer (Agilent Technologies, Santa Clara, CA, USA).

RNA sequencing libraries were constructed using the NEBNext Ultra RNA Library Preparation Kit (Illumina, San Diego, CA, USA) following the manufacturer’s protocol. Libraries were sequenced on an Illumina NovaSeq 6000 platform using paired-end 150-bp sequencing by OE Biotech Co., Ltd. (Shanghai, China). Raw sequencing reads were processed using Trimmomatic v0.39 to remove adaptor sequences and low-quality reads [34]. Clean reads were aligned to the maize B73 reference genome (RefGen_v4) using HISAT2 [35]. Differentially expressed genes (DEGs) were identified using DESeq2 according to |log2(fold change)| ≥ 1 and Benjamini–Hochberg-adjusted *p*-value (false discovery rate, FDR) < 0.05 [36,37].

### 2.4. Transcriptome Data Analysis

Principal component analysis (PCA) was performed using normalized gene expression matrices to evaluate sample relationships and biological reproducibility. Hierarchical clustering analysis was conducted to visualize global transcriptional patterns among genotypes and developmental stages. Gene Ontology (GO) enrichment analysis was performed using the clusterProfiler R package. Kyoto Encyclopedia of Genes and Genomes (KEGG) pathway enrichment analysis was conducted to identify biological pathways significantly enriched among DEGs [38]. Pathways with *p* < 0.05 were considered significantly enriched.

### 2.5. Weighted Gene Co-Expression Network Analysis (WGCNA)

Weighted gene co-expression network analysis (WGCNA) was performed using the WGCNA R package to identify gene co-expression modules associated with embryo dehydration dynamics [39]. Normalized gene expression profiles from all 36 embryo transcriptome samples were used for network construction, including four maize inbred lines, three developmental stages (35, 45, and 55 DAP), and three independent biological replicates for each genotype × developmental-stage combination.

After module detection and module merging, module eigengenes were calculated to summarize the expression profiles of the identified co-expression modules. For module–trait association analysis, AUDDC was used as the dehydration-related physiological trait. To ensure that the expression and physiological data were matched at the same experimental unit and to avoid treating repeated biological replicates with identical AUDDC values as independent trait observations, module eigengene values were averaged across the three biological replicates for each genotype × developmental-stage combination.

Thus, module–trait correlations were calculated using 12 genotype × developmental-stage observations. The corresponding AUDDC values were assigned according to the developmental interval corresponding to each transcriptome sampling stage. Specifically, samples collected at 35 DAP were matched with AUDDC values calculated for the 35–40 DAP interval, whereas samples collected at 45 and 55 DAP were matched with AUDDC values calculated for the 45–50 and 50–55 DAP intervals, respectively. Pearson correlation analysis was then performed between module eigengenes and the corresponding AUDDC values. Modules showing significant correlations with AUDDC were selected for subsequent functional enrichment and expression analyses.

### 2.6. Untargeted Metabolomic Profiling of Embryo Tissues

Untargeted metabolomic profiling was performed using embryo samples collected from the representative fast-dehydrating genotype HCL645 and slow-dehydrating genotype 911 at 35, 45, and 55 DAP. Approximately 100 mg of frozen embryo tissue was ground into a fine powder under liquid nitrogen. Metabolites were extracted using 80% methanol containing internal standards. The extracted samples were vortexed, sonicated, and centrifuged at 13,000 rpm for 10 min at 4 °C. The resulting supernatants were filtered through 0.22 μm membrane filters and transferred to LC-MS vials for subsequent analysis. Metabolomic profiling was conducted using ultra-high-performance liquid chromatography coupled with high-resolution mass spectrometry (UHPLC-MS). Raw mass spectrometry data were processed using Compound Discoverer 3.2 (Thermo Fisher Scientific, Waltham, MA, USA). Peak detection, alignment, and deconvolution were performed using MS-DIAL (Mass spectrometry-data independent analysis software, version 4.90). Metabolite annotation was performed by matching experimental spectra against mzCloud, mzVault, MassList, and ChemSpider databases based on accurate mass, retention time, and MS/MS fragmentation information.

### 2.7. Metabolomic Data Analysis

Principal component analysis (PCA) was performed using normalized metabolite abundance matrices to evaluate overall metabolic variation among samples. Partial least squares discriminant analysis (PLS-DA) was used as an exploratory supervised multivariate approach to visualize metabolomic separation between HCL645 and 911. Metabolomic data were Pareto-scaled before PLS-DA. The number of latent components was selected by 7-fold cross-validation. Model reliability was evaluated by 200 permutation tests. The parameters R^2^X (cum), R^2^Y (cum), Q^2^ (cum), R^2^ intercept, and Q^2^ intercept were recorded. A negative Q^2^ intercept was considered indicative of no obvious overfitting. Differentially accumulated metabolites (DAMs) were identified using a combination of multivariate, fold-change, and multiple-testing-adjusted criteria. Metabolites with a variable importance in projection (VIP) score > 1, an absolute log2 fold change (|log2FC|) ≥ 1, and a Benjamini–Hochberg-adjusted q-value < 0.05 were defined as DAMs. Raw *p*-values are retained in Supplementary Tables S7 and S8 for transparency but were not used as the final significance criterion for DAM identification. Hierarchical clustering and heatmap visualization were performed using R software (version 4.3.2). KEGG enrichment analysis was performed to identify metabolic pathways significantly associated with DAMs.

### 2.8. Integrated Transcriptomic and Metabolomic Analysis

To investigate pathway-level convergence between transcriptomic and metabolic changes associated with embryo dehydration variation, DEGs and FDR-controlled DAMs were mapped independently to KEGG pathways. Pathways represented by both DEGs and DAMs were identified as pathway-level convergence and considered candidate biological processes associated with contrasting embryo dehydration phenotypes. No pairwise gene–metabolite correlation analysis was performed. Accordingly, no correlation method, number of observations for correlation, significance threshold for correlation, or multiple-testing correction for correlation is reported. The integrated results were interpreted as pathway-level associations and coordinated molecular patterns rather than as direct evidence of regulatory interactions or causal gene–metabolite relationships.

### 2.9. Statistical Analysis

The physiological experiment was conducted using a factorial design in which four maize genotypes were evaluated across multiple developmental stages. For each genotype × developmental-stage combination, three independent biological replicates were analyzed. Each biological replicate represented an independently collected sample of kernels or dissected tissues harvested from independently sampled plants or ears. Agronomic traits were evaluated using five independent biological replicates (*n* = 5). Kernel-, embryo-, and endosperm-related physiological measurements were performed using three independent biological replicates (*n* = 3). Transcriptomic and metabolomic analyses included three independent biological replicates for each genotype × developmental stage combination. Different developmental stages were sampled independently rather than repeatedly measured from the same experimental units; therefore, measurements obtained at different developmental stages were treated as independent observations.

For physiological traits measured across genotypes and developmental stages, the effects of genotype, developmental stage, and their interaction were evaluated using two-way analysis of variance (ANOVA). The statistical model included genotype and developmental stage as fixed factors and the genotype × developmental-stage interaction. When significant main effects or interactions were detected, multiple comparisons among genotype × developmental-stage means were performed using Tukey’s honestly significant difference (HSD) test. For traits evaluated separately at individual developmental stages, one-way ANOVA followed by Tukey’s HSD test was used to compare genotypes. For AUDDC, one-way ANOVA was used to evaluate genotype effects. Data are presented as means ± standard deviation (SD), and the number of independent biological replicates is indicated in the corresponding figure legends or table notes.

Differentially expressed genes were identified using |log2FC| ≥ 1 and an FDR-adjusted q-value < 0.05. DAMs were identified using VIP > 1, |log2FC| ≥ 1, and a Benjamini–Hochberg-adjusted q-value < 0.05. Pearson correlation analysis was used to evaluate associations among dehydration-related and agronomic traits. Statistical analyses were performed using GraphPad Prism 9.0, Microsoft Excel 2021, and R software (version 4.3.2). Transcriptomic and metabolomic datasets were integrated at the pathway level. Significantly changed genes and metabolites were independently mapped to biological pathways, and pathways represented by changes in both datasets were considered candidate processes associated with contrasting embryo dehydration phenotypes. Pairwise gene–metabolite correlations were not used as evidence for direct regulatory relationships.

## 3. Results

### 3.1. Variation in Kernel, Endosperm, and Embryo Moisture Content and Dehydration Rate During Maize Seed Maturation

**Figure 1:**
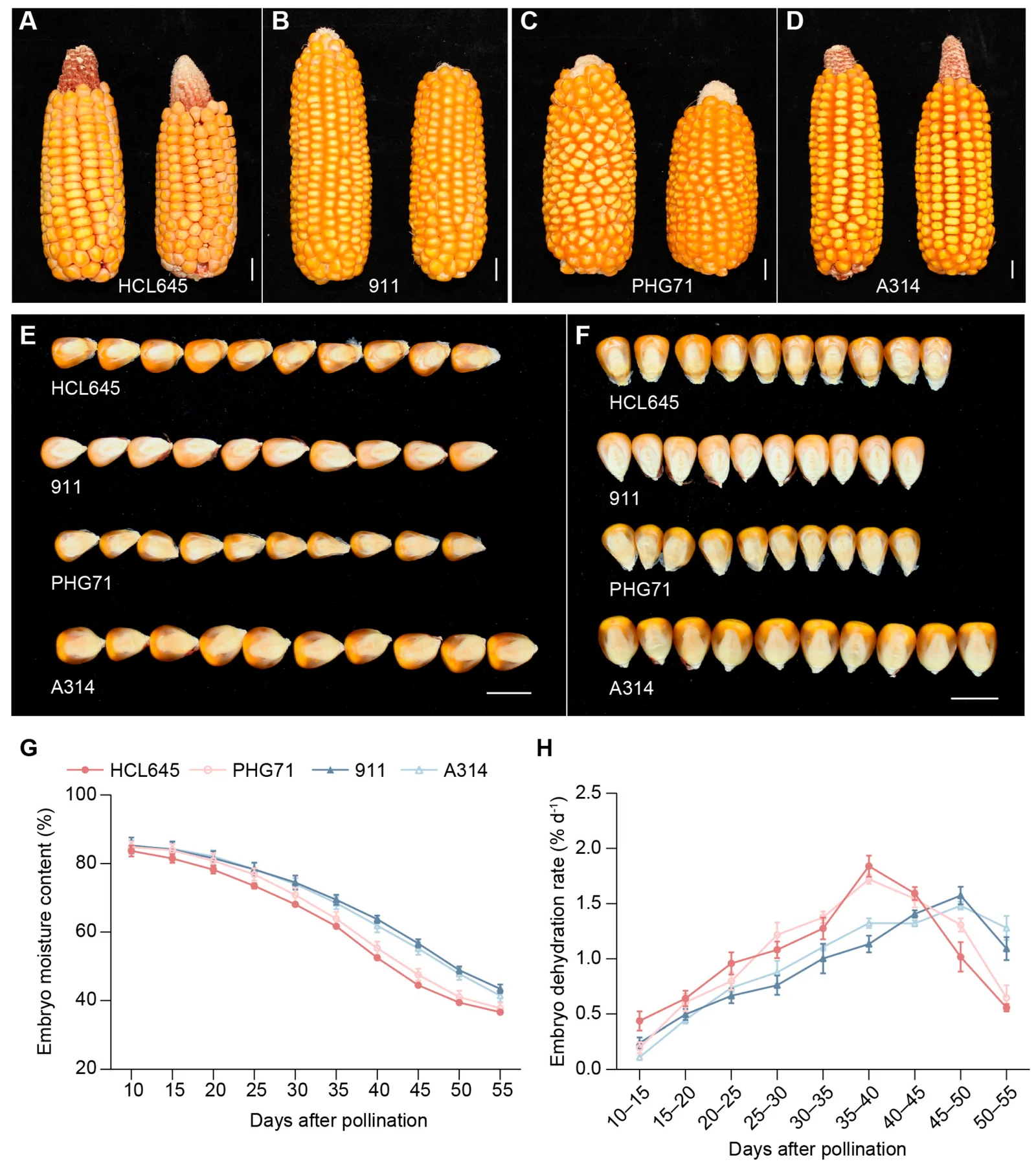
Developmental changes in moisture content and dehydration rate in maize kernels, endosperms, and embryos during kernel maturation. (**A**–**D**) Morphological comparison of representative mature ears from fast-dehydrating (FDR; HCL645 and PHG71) and slow-dehydrating (SDR; 911 and A314) maize inbred lines. (**E**) Side view of individual kernels arranged longitudinally. (**F**) Front view (embryo side) of the corresponding kernels. Scale bars: 2 cm in (**A**–**D**) and 1 cm in (**E**,**F**). (**G**) Changes in embryo moisture content from 10 to 55 days after pollination (DAP). (**H**) Changes in embryo dehydration rate during successive developmental intervals. Data in (**G**,**H**) represent means ± standard deviation (SD). Data are presented as means ± SD (n = 3 independent biological replicates). Genotype, developmental stage, and their interaction were evaluated by two-way ANOVA, followed by Tukey’s HSD test for multiple comparisons where appropriate.

To characterize physiological variation associated with maize kernel dehydration, four representative inbred lines (HCL645, PHG71, 911, and A314) with contrasting dehydration capacities were selected from a previously established association panel comprising 309 maize inbred lines [31]. The four lines were classified according to their dehydration performance into fast-dehydrating (FDR; HCL645 and PHG71) and slow-dehydrating (SDR; 911 and A314) groups. Significant differences in ear and kernel morphology were observed among the four inbred lines (Figure 1; Table S1A). The ears of PHG71 and 911 had greater diameters than those of HCL645 and A314, whereas HCL645 and 911 had shorter ears than PHG71 and A314 (Figure 1A–D; Table S1A). Among the four inbred lines, A314 exhibited the greatest kernel length and width, followed by HCL645 and 911, whereas PHG71 had the smallest kernels (Figure 1E,F; Table S1A).

Dynamic changes in moisture content (MC) and dehydration rate (DR) were characterized in whole kernels, endosperms, and embryos from 10 to 55 DAP (Figure 1G,H and Figure S1A–D; Table S1A). All three tissues underwent progressive water loss during maturation; however, the extent and temporal patterns of dehydration differed among genotypes and tissues. Compared with the SDR lines, the FDR lines exhibited more rapid dehydration and lower moisture contents across all kernel tissues, particularly during late maturation (Figure S1A,B; Table S1A). At 55 DAP, the kernel MCs of HCL645 and PHG71 decreased to 21.23% and 21.91%, respectively, whereas those of 911 and A314 remained considerably higher at 32.51% and 35.19%, respectively (Figure S1A; Table S1A). Consistently, the kernel DRs of HCL645 and PHG71 reached 1.44 and 1.42% d^−1^, respectively, exceeding those of 911 and A314 (1.20 and 1.11% d^−1^, respectively) (Figure S1B; Table S1A). The consistently lower AUDDC values observed in FDR lines further supported their greater dehydration performance during kernel maturation (Table S1A).

In both the FDR and SDR inbred lines, the temporal patterns of endosperm MC and DR generally resembled those observed for whole kernels, indicating a close association between endosperm dehydration dynamics and overall kernel dry-down (Figure S1C,D; Table S1A). Notably, embryo dehydration also exhibited developmental patterns closely associated with whole-kernel dehydration. Although embryos maintained higher absolute moisture contents than whole kernels and endosperms throughout development, substantial differences in embryo dehydration capacity were observed among the four inbred lines (Figure 1G; Table S1A). At maturity, embryos of HCL645 and PHG71 exhibited lower moisture contents (36.64% and 37.78%, respectively) and higher dehydration rates (1.04 and 1.05% d^−1^, respectively) than those of 911 and A314 (41.42–43.40% and 0.93–0.97% d^−1^, respectively) (Figure 1G,H; Table S1A).

Correlation analysis further revealed close associations between moisture-related traits of individual kernel tissues and whole-kernel dehydration traits. Kernel MC was positively correlated with endosperm MC (*r* = 0.83) and embryo MC (*r* = 0.84), whereas kernel dehydration rate was positively correlated with endosperm DR (*r* = 0.78) and embryo DR (*r* = 0.80) (Figure S2). These relationships indicate that dehydration dynamics of the embryo and endosperm were closely associated with overall kernel dry-down. Embryo MC was also associated with several kernel morphological and yield-related traits, including kernel weight per ear (r = 0.69), kernel width (*r* = 0.48), and kernel thickness (*r* = 0.61). In contrast, kernel DR was negatively associated with kernel weight per ear (*r* = −0.90), ear diameter (*r* = −0.76), and 100-kernel weight (*r* = −0.80) (Figure S2).

To evaluate whether the responses of moisture-related traits across developmental stages differed among genotypes, two-way ANOVA was performed with genotype, developmental stage (DAP), and their interaction as fixed factors. For all three tissues (kernel, endosperm, and embryo), genotype, developmental stage, and genotype × developmental stage interaction had highly significant effects on both moisture content and dehydration rate (all *p* < 0.001; Table S1B). Specifically, for kernel moisture content, the effects of genotype (F = 246.90, df = 3, *p* < 0.001), developmental stage (F = 3450.54, df = 9, *p* < 0.001), and their interaction (F = 17.92, df = 27, *p* < 0.001) were all significant. Similar significant effects were observed for endosperm and embryo moisture content and for dehydration rate in all tissues (Table S1B). For AUDDC, one-way ANOVA revealed significant genotype effects for kernel (F = 104.47, df = 3, *p* < 0.001), endosperm (F = 20.21, df = 3, *p* < 0.001), and embryo (F = 32.49, df = 3, *p* < 0.001). These results indicate that the four maize inbred lines differed significantly in their moisture-related physiological traits and that the patterns of change across developmental stages were genotype-dependent, as evidenced by the significant genotype × developmental stage interactions.

Together, these physiological associations indicate that variation in embryo dehydration occurs in parallel with broader differences in kernel dehydration and agronomic characteristics among the four selected genotypes. However, because the embryo and endosperm are structural components of the kernel and comparable associations were observed for both tissues, these correlations alone do not quantify the relative contribution of tissue-specific water loss to whole-kernel dehydration.

### 3.2. Transcriptome Profiling Reveals Developmental- and Genotype-Dependent Expression Changes During Embryo Dehydration

To characterize developmental transcriptomic changes associated with contrasting embryo dehydration phenotypes, RNA sequencing was performed on embryos collected from four maize inbred lines (HCL645, PHG71, 911, and A314) at 35, 45, and 55 DAP. A total of 36 RNA-seq libraries were generated, representing four genotypes, three developmental stages, and three independent biological replicates for each genotype × developmental-stage combination. PCA and hierarchical clustering showed clear separation among developmental stages and genotypes, while biological replicates generally clustered closely together, indicating good consistency among the transcriptomic samples (Figure S3).

**Figure 2:**
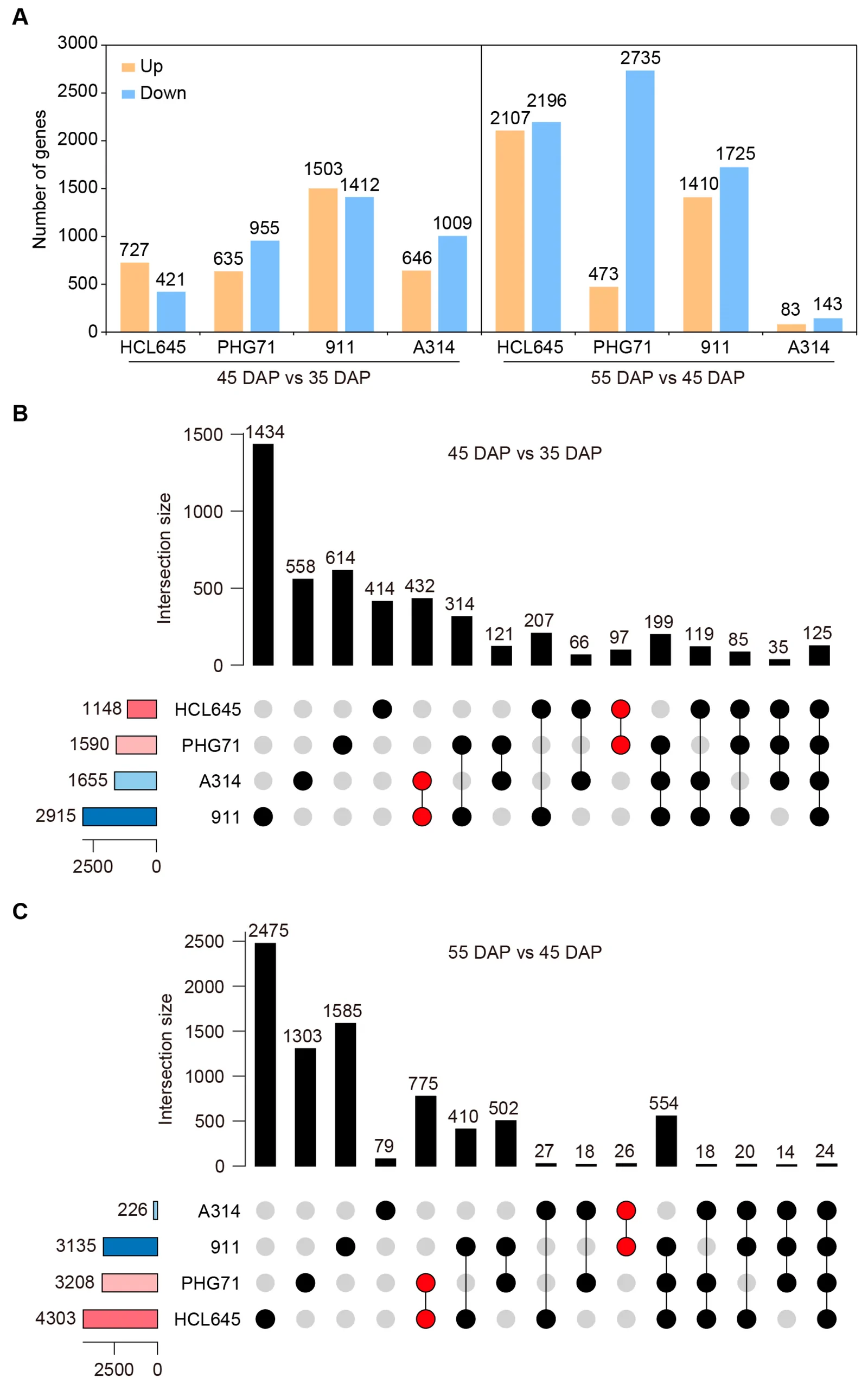
DEGs identified during embryo maturation in four maize inbred lines with contrasting dehydration phenotypes. (**A**) Numbers of upregulated (orange) and downregulated (blue) DEGs identified in HCL645, PHG71, 911, and A314 embryos during the 45 vs. 35 DAP (**left**) and 55 vs. 45 DAP (**right**) developmental transitions. DEGs were defined using |log2(fold change)| ≥ 1 and an FDR-adjusted *q*-value < 0.05. (**B**,**C**) UpSet plots showing intersections of DEGs among the four genotypes during the 45 vs. 35 DAP (**B**) and 55 vs. 45 DAP (**C**) transitions. Horizontal bar charts represent the total number of DEGs identified in each genotype, whereas vertical bar charts indicate the sizes of individual DEG intersection sets. Red circles highlight the intersections of DEGs shared between the two FDR genotypes or between the two SDR genotypes.

DEGs were identified between consecutive developmental stages using the criteria of |log2(fold change)| ≥ 1 and an FDR-adjusted *q*-value < 0.05. During the early developmental transition (45 vs. 35 DAP), 1148 DEGs (727 upregulated and 421 downregulated), 1590 DEGs (635 upregulated and 955 downregulated), 2915 DEGs (1503 upregulated and 1412 downregulated), and 1655 DEGs (646 upregulated and 1009 downregulated) were identified in HCL645, PHG71, 911, and A314, respectively (Figure 2A and Figure S4; Table S2). During the late developmental transition (55 vs. 45 DAP), the number of DEGs increased substantially in HCL645, PHG71, and 911, with 4303 DEGs (2107 upregulated and 2196 downregulated), 3208 DEGs (473 upregulated and 2735 downregulated), and 3135 DEGs (1410 upregulated and 1725 downregulated), respectively (Figure 2A and Figure S5; Table S3). In contrast, A314 exhibited a substantially smaller number of transcriptional changes, with only 226 DEGs (83 upregulated and 143 downregulated) identified during the same developmental transition (Figure 2A and Figure S5; Table S3).

UpSet analysis was subsequently performed to examine shared and genotype-specific transcriptional changes among the four genotypes during embryo maturation (Figure 2B,C). During the early developmental transition (45 vs. 35 DAP), the numbers of genotype-specific DEGs in HCL645, PHG71, 911, and A314 were 414, 614, 1413, and 558, respectively. In addition, 97 and 432 DEGs were shared between the two FDR genotypes and between the two SDR genotypes, respectively (Figure 2B). During the late developmental transition (55 vs. 45 DAP), genotype-specific DEGs increased markedly in HCL645, PHG71, and 911, reaching 2475, 1303, and 1585, respectively, whereas only 79 genotype-specific DEGs were detected in A314. Meanwhile, 775 and 26 were shared between the two FDR genotypes and between the two SDR genotypes, respectively (Figure 2C).

Overall, the transcriptomic analysis revealed pronounced developmental- and genotype-dependent variation in embryo gene expression. The contrasting patterns of DEG overlap between the FDR and SDR genotype groups provide a basis for subsequent analyses of shared and genotype-specific molecular processes associated with differential embryo dehydration.

### 3.3. Functional Enrichment Analysis of Shared DEGs During Embryo Maturation

To further characterize the biological processes represented by genes showing coordinated expression changes within each dehydration group, Gene Ontology (GO) enrichment analysis was performed using DEGs shared between the two FDR genotypes and between the two SDR genotypes during the early and late developmental transitions.

**Figure 3:**
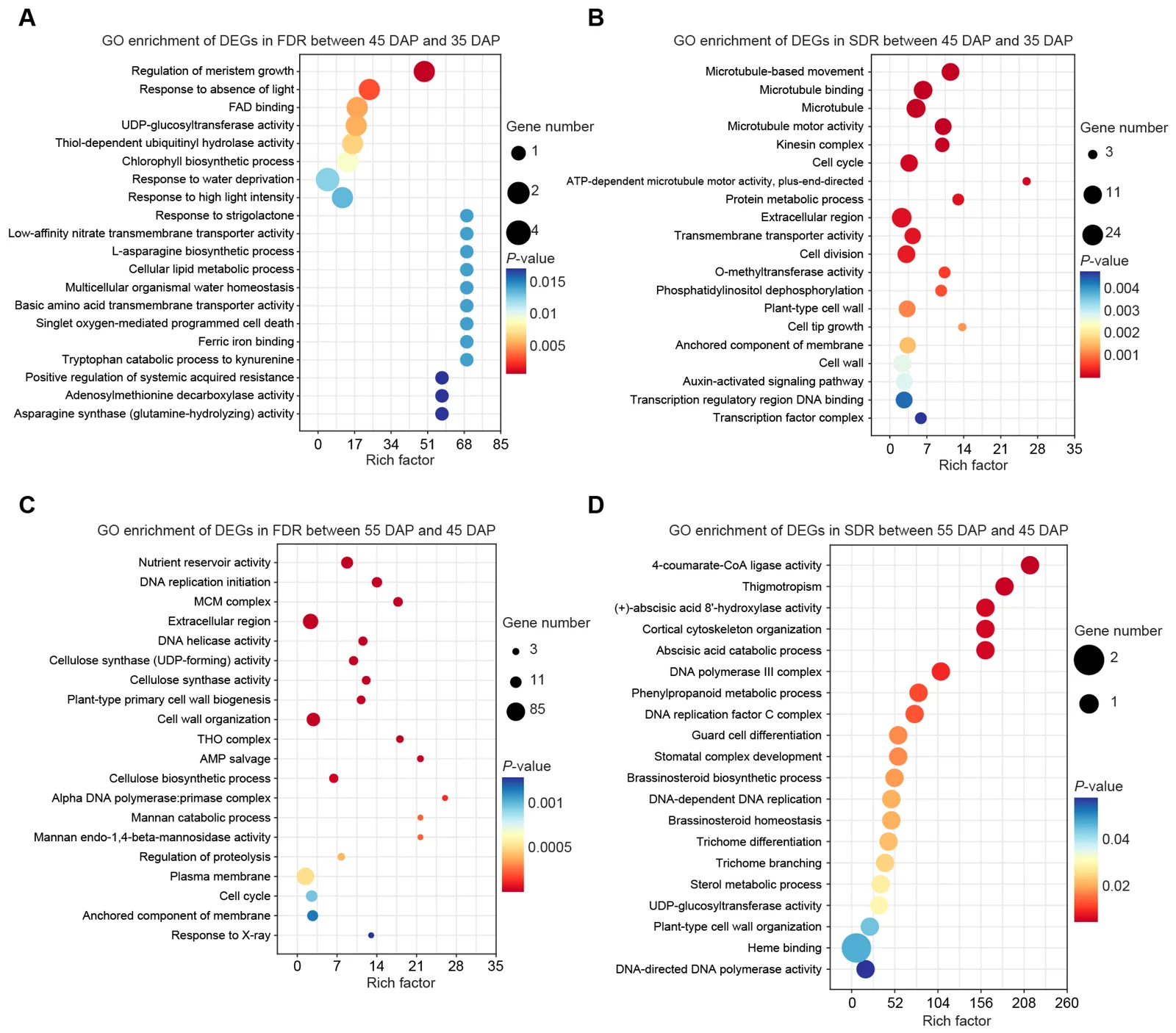
GO enrichment analysis of shared DEGs in maize embryos during two developmental transitions. (**A**,**B**) GO enrichment analysis of DEGs shared between the two fast-dehydrating (FDR) and two slow-dehydrating (SDR) genotypes, respectively, during the early developmental transition (45 vs. 35 DAP). (**C**,**D**) GO enrichment analysis of DEGs shared between the two FDR and two SDR genotypes, respectively, during the late developmental transition (55 vs. 45 DAP). DAP, days after pollination; FDR, fast dehydration rate; SDR, slow dehydration rate.

During the early developmental transition (45 vs. 35 DAP), DEGs shared between the two FDR genotypes were significantly enriched in biological processes related to metabolic regulation, macromolecule biosynthesis, protein modification, and the regulation of cellular processes (Figure 3A). These enrichment patterns indicate that genes showing coordinated expression changes in the two FDR genotypes were associated with multiple metabolic and developmental processes during early embryo maturation. In contrast, DEGs shared between the two SDR genotypes were primarily enriched in biological processes related to cellular organization, carbohydrate metabolism, stress responses, and the regulation of biological processes (Figure 3B), indicating distinct patterns of shared transcriptional changes during the same developmental transition.

During the late developmental transition (55 vs. 45 DAP), DEGs shared between the two FDR genotypes were significantly enriched in GO categories related to cell wall organization, extracellular region-related functions, transport processes, and responses to environmental stimuli (Figure 3C). In contrast, the relatively small set of DEGs shared between the two SDR genotypes was enriched in GO categories related to oxidation–reduction processes, secondary metabolic processes, carbohydrate metabolism, and cellular responses to stress (Figure 3D).

Collectively, these enrichment patterns indicate that the biological processes represented by shared DEGs differed between the FDR and SDR genotype groups and varied across developmental transitions. During the late developmental transition, DEGs shared between the two FDR genotypes were predominantly represented by categories related to structural organization, transport, and responses to environmental stimuli, whereas DEGs shared between the two SDR genotypes were enriched in categories related to redox processes and metabolism. These differences describe distinct patterns of coordinated gene-expression change associated with contrasting dehydration phenotypes and identify candidate biological processes for further investigation.

### 3.4. Identification of Gene Co-Expression Modules Associated with Embryo Dehydration Traits Using WGCNA

**Figure 4:**
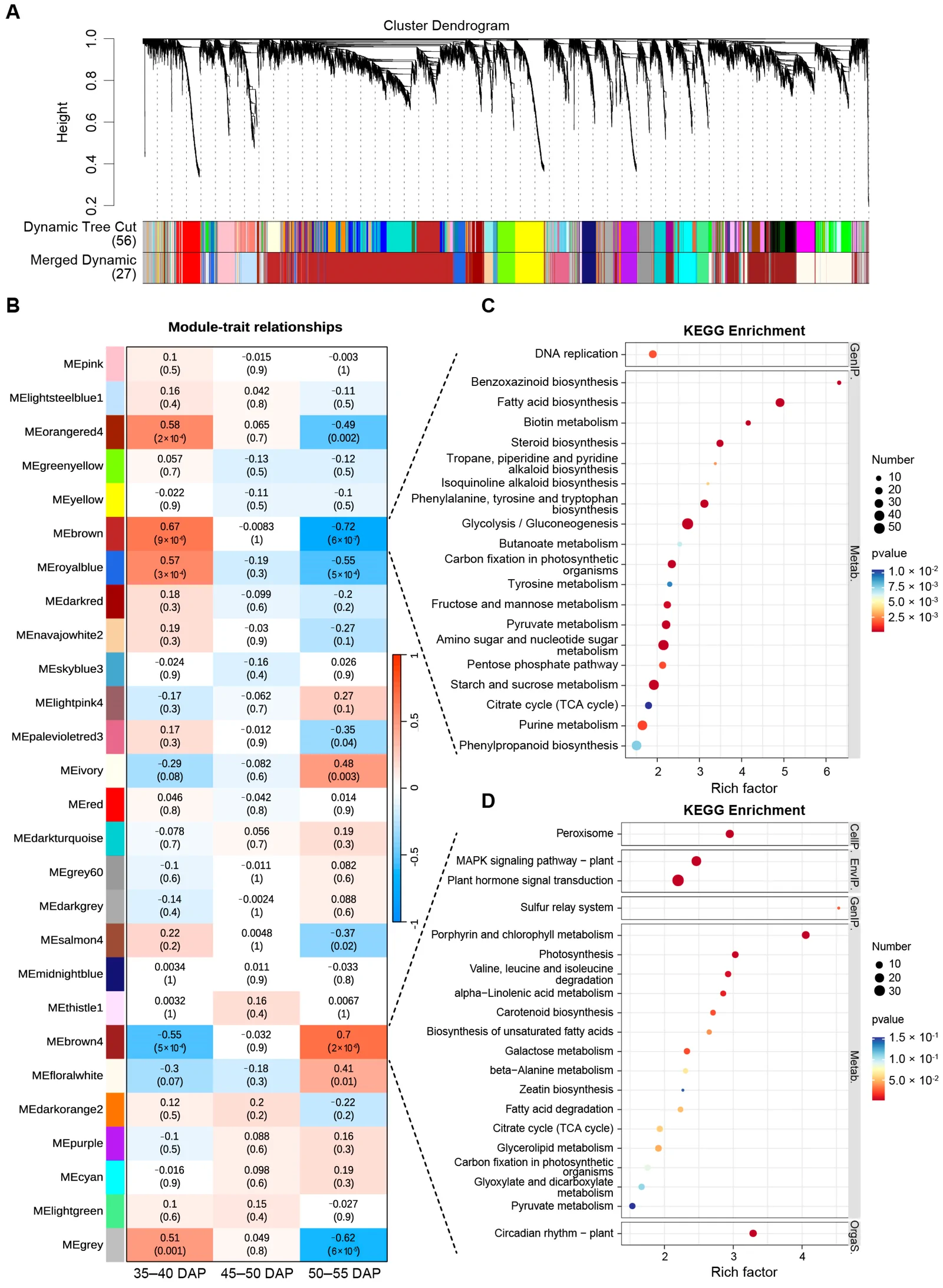
WGCNA and KEGG pathway enrichment of gene co-expression modules associated with embryo dehydration traits. (**A**) Hierarchical clustering dendrogram showing gene co-expression patterns and the identification of 56 initial modules, which were subsequently merged into 27 modules based on eigengene similarity. (**B**) Module–trait relationship heatmap showing Pearson correlation coefficients between module eigengenes and AUDDC across 12 genotype × developmental-stage observations. Module eigengene values were averaged across the three biological replicates for each genotype × developmental-stage combination. Transcriptome samples collected at 35, 45, and 55 DAP were matched with AUDDC values calculated for the 35–40, 45–50, and 50–55 DAP intervals, respectively. (**C**,**D**) KEGG enrichment analyses of genes within the MEbrown (**C**) and MEbrown4 (**D**), respectively. These modules were significantly associated with embryo dehydration traits in (**B**). Bubble size represents the number of genes enriched in each pathway, and color intensity indicates the significance level.

To identify gene co-expression patterns associated with embryo dehydration dynamics, WGCNA was performed using normalized gene-expression profiles from all 36 embryo transcriptome samples. These samples comprised four maize inbred lines sampled at 35, 45, and 55 DAP with three independent biological replicates per genotype × developmental-stage combination. A total of 56 initial co-expression modules were identified and subsequently merged into 27 modules based on eigengene similarity (Figure 4A). For the module–trait association analysis, the AUDDC was used as the dehydration-related physiological trait. To avoid treating biological replicates sharing identical physiological trait values as independent observations, module eigengene values were averaged across the three biological replicates for each genotype × developmental-stage combination. Module–trait correlations were therefore calculated using 12 genotype × developmental-stage observations. Transcriptome samples collected at 35, 45, and 55 DAP were matched with AUDDC values calculated for the 35–40, 45–50, and 50–55 DAP intervals, respectively. Pearson correlation analysis identified two co-expression modules, MEbrown and MEbrown4, that were significantly associated with embryo dehydration dynamics (Figure 4B).

The MEbrown module showed a significant positive correlation with AUDDC during early embryo maturation (35–40 DAP; *r* = 0.67, *p* = 9 × 10^−6^), and a significant negative correlation during late maturation (50–55 DAP; *r* = −0.72, *p* = 6 × 10^−7^). KEGG enrichment analysis showed that genes in the MEbrown module were enriched in multiple metabolic pathways, including benzoxazinoid biosynthesis, fatty acid biosynthesis, starch and sucrose metabolism, steroid biosynthesis, phenylalanine, tyrosine and tryptophan biosynthesis, glycolysis/gluconeogenesis, fructose and mannose metabolism, pyruvate metabolism, the pentose phosphate pathway, and the citrate cycle (TCA cycle) (Figure 4C). These pathways encompass carbon and energy metabolism, energy metabolism, and lipid-associated processes, indicating that the MEbrown module contains genes involved in multiple metabolic processes that vary during embryo maturation and are correlated with differences in dehydration dynamics.

The MEbrown4 module also showed significant associations with dehydration dynamics, with a significant negative correlation with AUDDC at 35–40 DAP (*r* = −0.55, *p* = 5 × 10^−4^) and a strong positive correlation at 50–55 DAP (*r* = 0.70, *p* = 2 × 10^−6^). KEGG enrichment analysis indicated that genes in this module were significantly enriched in pathways related to environmental responses, signal transduction, and metabolism, including plant–pathogen interaction, the MAPK signaling pathway, plant hormone signal transduction, peroxisome-related metabolism, α-linolenic acid metabolism, fatty acid degradation, and citrate cycle (TCA cycle) (Figure 4D). These enrichment patterns indicate that the MEbrown4 module contains genes associated with hormone signaling, MAPK-related signaling, stress responses, and lipid-associated metabolic processes. The associations of this module with AUDDC identify these biological processes as candidate molecular processes associated with developmental and genotype-dependent variation in embryo dehydration that warrant further functional investigation.

### 3.5. Candidate Metabolic and Signaling Processes Associated with Embryo Dehydration Revealed by Dehydration-Associated Gene Modules

**Figure 5:**
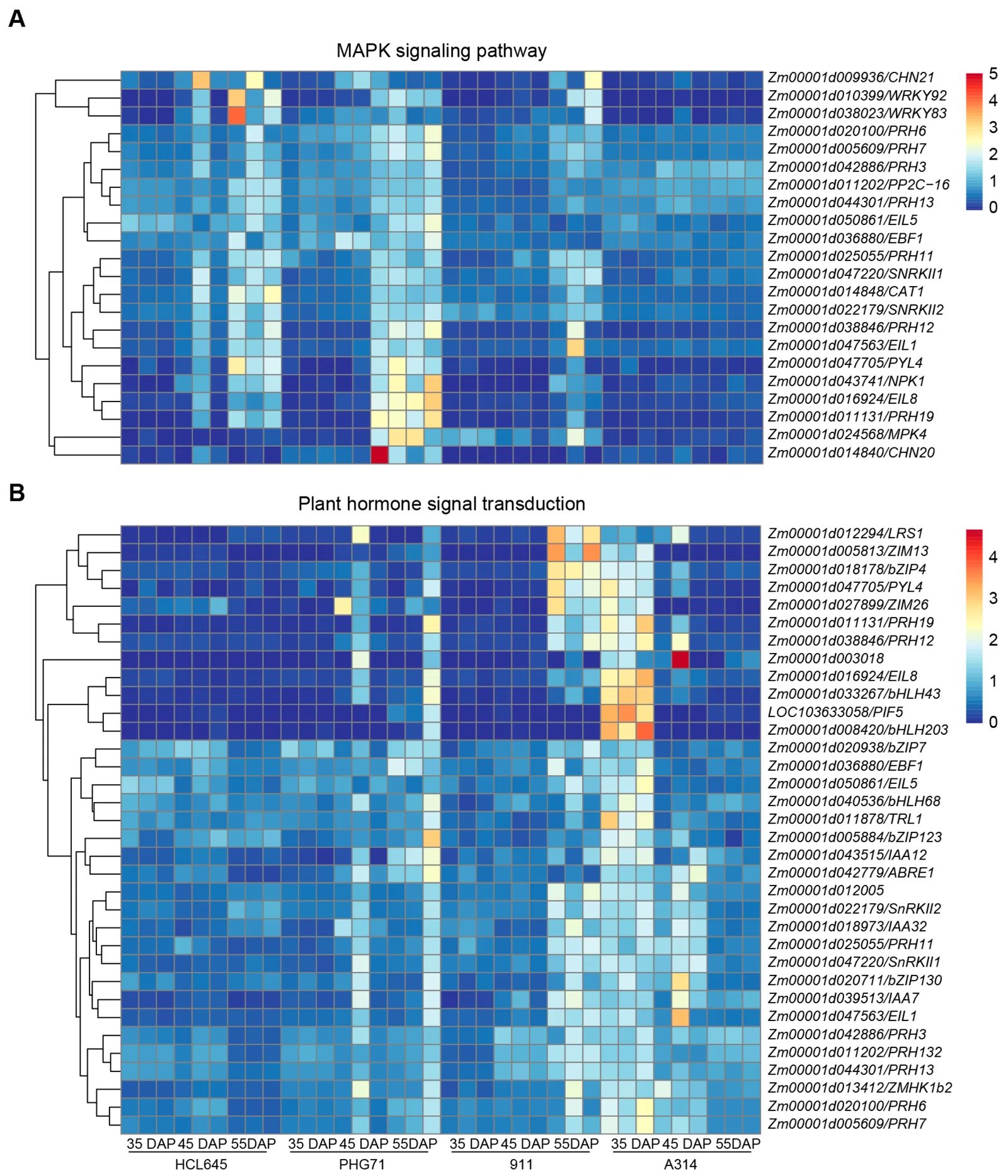
Expression patterns of representative genes involved in MAPK signaling and plant hormone signal transduction within the dehydration-associated MEbrown4 module. (**A**) Heatmap showing the expression patterns of genes associated with the MAPK signaling pathway that were enriched in the MEbrown4 module. (**B**) Heatmap showing the expression patterns of genes associated with plant hormone signal transduction within the same MEbrown4 module. Rows represent individual genes, and columns represent biological replicates from HCL645, PHG71, 911, and A314 embryos collected at 35, 45, and 55 DAP. Color intensity represents relative gene expression levels, with red indicating relatively higher expression and blue indicating relatively lower expression.

To further characterize candidate molecular processes associated with embryo dehydration, we examined representative genes within the dehydration-associated MEbrown and MEbrown4 modules. Within the MEbrown module, genes associated with membrane lipid metabolism and energy metabolism displayed distinct expression patterns between FDR and SDR embryos (Figure 5 and Figure S6; Tables S4 and S5).

Several genes annotated to de novo fatty acid biosynthesis, including *ACC1* (acetyl-CoA carboxylase 1), *ACC2*, *FAB1* (3-ketoacyl-ACP synthase), *FAT2* (fatty acyl-ACP thioesterase), members of the *SACD* gene family (*SACD1/2/5/7*), and *LACS2* (long-chain acyl-CoA synthetase 2), exhibited differential expression patterns among genotypes with contrasting dehydration phenotypes (Figure S6A; Table S4). These expression patterns identify fatty acid biosynthesis as a candidate metabolic process associated with developmental and genotype-dependent variation in embryo dehydration. In addition, numerous genes involved in glycolysis, including *PFK1* (phosphofructokinase), *PYK1/PYK3* (pyruvate kinase), *HEX4/HEX5/HEX9* (hexokinase), *ENO1* (enolase), *PGK2/PGK3* (phosphoglycerate kinase), *TPI2/TPI4/TPI5* (triosephosphate isomerase), *ALD1/ALD2* (fructose-bisphosphate aldolase), *ADH1/ADH2* (alcohol dehydrogenase), and *PDC3* (pyruvate decarboxylase), showed pronounced developmental stage-dependent expression patterns (Figure S6B; Table S4). Together, these observations indicate substantial developmental variation in the expression of genes involved in carbohydrate and energy metabolism and identify these metabolic processes as candidates associated with contrasting embryo dehydration phenotypes.

Within the MEbrown4 module, genes associated with hormone signaling and MAPK cascades were further examined because of their potential involvement in integrating developmental signals and environmental stress responses (Figure 5A; Table S5). Components of ABA signaling, including *PYL4* (ABA receptor), *PP2C-16/PP2C132* (protein phosphatase 2C), and *SnRKII1/SnRKII2* (SNF1-related protein kinase 2), as well as components of ethylene signaling, including *EIL1, EIL5, EIL8* (ethylene-insensitive-like transcription factors), and *EBF1* (EIN3-binding F-box protein), exhibited distinct expression patterns between FDR and SDR embryos (Figure 5B; Table S5). These observations identify ABA- and ethylene-associated signaling components as candidate molecular processes associated with differences in embryo dehydration phenotypes.

Several ABA-responsive transcription factors, including *bZIP7*, *bZIP123*, *ABRE1*, and related *ABF*-type regulators, were also represented in this module, further identifying ABA-associated transcriptional regulation as a candidate process associated with embryo maturation and dehydration dynamics (Figure 5B; Table S5). The differential expression of these genes indicates genotype- and developmental stage-dependent variation in ABA-associated transcriptional patterns but does not, by itself, establish a causal role for ABA signaling in embryo dehydration. In addition, the enrichment of MAPK signaling and the differential expression of MAPK-related genes indicate another candidate process associated with developmental and genotype-dependent variation in embryo dehydration.

Collectively, these expression patterns indicate that the MEbrown and MEbrown4 modules contain genes associated with lipid and carbohydrate metabolism, MAPK signaling, and plant hormone signal transduction. These pathways represent candidate molecular processes associated with developmental and genotype-dependent variation in embryo dehydration and provide targets for future functional investigation.

### 3.6. Metabolomic Patterns and Pathway-Level Associations in HCL645 and 911 Embryos During Kernel Maturation

**Figure 6:**
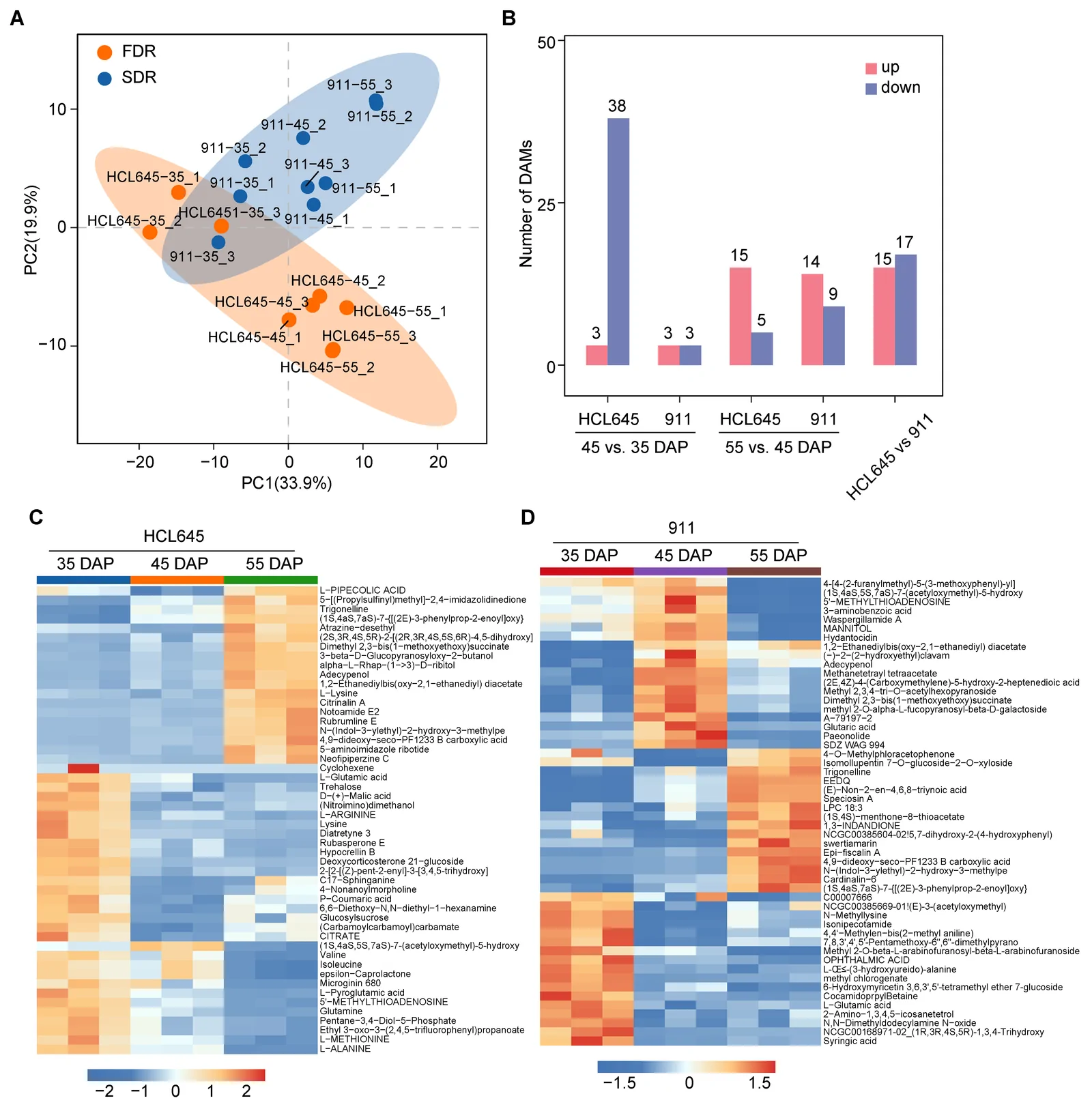
Untargeted metabolomic profiling of HCL645 and 911 embryos during kernel maturation. (**A**) Principal component analysis (PCA) score plot of embryo metabolomic profiles from the fast-dehydrating genotype HCL645 (FDR) and the slow-dehydrating genotype 911 (SDR) at 35, 45, and 55 DAP. PC1 and PC2 explained 33.9% and 19.9% of the total variance, respectively. (**B**) Numbers of DAMs with increased and decreased abundance identified in HCL645 and 911 embryos during the 45 vs. 35 DAP and 55 vs. 45 DAP developmental transitions, as well as in the between-genotype comparison between HCL645 and 911. Red and blue bars represent DAMs with increased and decreased abundance, respectively. (**C**,**D**) Heatmaps showing relative abundance patterns (z-scores) of DAMs in HCL645 (**C**) and 911 (**D**) embryos across 35, 45, and 55 DAP.

To characterize metabolomic variation associated with contrasting embryo dehydration phenotypes, untargeted metabolomic profiling was performed on embryos from the fast-dehydrating genotype HCL645 and the slow-dehydrating genotype 911 at 35, 45, and 55 DAP. A total of 4998 metabolic features were detected, including 3046 annotated metabolites, providing a broad dataset for examining developmental changes in embryo metabolism during kernel maturation (Table S6). PCA revealed clear separation of embryo metabolomic profiles according to both genotype and developmental stage, with PC1 and PC2 explaining 33.9% and 19.9% of the total variance, respectively (Figure 6A). Biological replicates within each genotype × developmental-stage group generally clustered together, supporting the reproducibility of the metabolomic profiles. PLS-DA showed clear separation between HCL645 and 911 embryos (Figure S7A). The model had R^2^X (cum) = 0.585, R^2^Y (cum) = 0.964, and Q^2^ (cum) = 0.806. Permutation testing (200 permutations) yielded an R^2^ intercept of 0.658 and a Q^2^ intercept of −1.708 (*p* < 0.05; Figure S7B), indicating that the model was reliable and did not show obvious overfitting.

To account for multiple testing arising from the large number of metabolites examined, DAMs were defined using multivariate, fold-change, and multiple-testing-adjusted statistical criteria. Metabolites with VIP > 1, |log2FC| ≥ 1, and a Benjamini–Hochberg-adjusted q-value < 0.05 were defined as DAMs (Tables S7 and S8). Raw *p*-values were retained in the Supplementary Tables but were not used as the final significance criterion for DAM identification. The resulting FDR-controlled DAM sets were subsequently used to characterize developmental and genotype-associated changes in metabolite abundance. Both genotypes exhibited statistically supported changes in metabolite abundance during embryo development, although the number and direction of DAMs differed between genotypes and across developmental transitions (Figure 6B; Tables S7 and S8). In HCL645, 41 DAMs were identified during the 45 vs. 35 DAP transition, including 38 with decreased abundance and 3 with increased abundance. During the 55 vs. 45 DAP transition, 20 DAMs were identified, including 15 with increased abundance and 5 with decreased abundance. In 911, 6 DAMs were identified during the 45 vs. 35 DAP transition, with 3 showing increased abundance and 3 showing decreased abundance, whereas 23 DAMs were identified during the 55 vs. 45 DAP transition, including 14 with increased abundance and 9 with decreased abundance. Comparisons between HCL645 and 911 also identified 32 DAMs, including 15 with higher abundance and 17 with lower abundance in the specified comparison (Figure 6B).

Hierarchical clustering and heatmap visualization further revealed genotype- and developmental-stage-dependent patterns of DAM abundance across 35, 45, and 55 DAP (Figure 6C,D). The DAMs represented in the heatmaps consisted predominantly of organic acids and related compounds, together with amino acid derivatives and fatty acid-related metabolites. Several metabolites were associated with central carbon and organic acid metabolism, including intermediates related to the tricarboxylic acid (TCA) cycle and short-chain oxo- and hydroxy acids. These patterns indicate that embryo maturation in HCL645 and 911 was accompanied by differences in the abundance of metabolites associated with organic acid, amino acid, and lipid-related metabolism.

Pathway enrichment analysis of the FDR-controlled DAMs identified several significantly enriched metabolic categories (Figure S8). These included ABC transporters, glyoxylate and dicarboxylate metabolism, and multiple amino acid metabolism-related pathways, including alanine, aspartate and glutamate metabolism, arginine biosynthesis, cysteine and methionine metabolism, D-amino acid metabolism, and aminoacyl-tRNA biosynthesis. Pathways associated with secondary metabolite biosynthesis, including glucosinolate biosynthesis and tropane, piperidine and pyridine alkaloid biosynthesis, were also represented among the enriched categories. The enrichment of ABC transporter-related annotations indicated the representation of transport-associated processes among the pathways associated with the DAMs, whereas enrichment of glyoxylate and dicarboxylate metabolism was consistent with the prominence of organic acid-related metabolites in the differential accumulation profiles.

Collectively, the metabolomic analysis revealed developmental- and genotype-associated differences in the abundance of metabolites related primarily to organic acid metabolism, amino acid metabolism, transport-associated processes, and secondary metabolism in HCL645 and 911 embryos. Together with the transcriptomic results, these findings identify candidate biological processes associated with contrasting embryo dehydration phenotypes. However, because metabolomic profiling was conducted using only one fast-dehydrating genotype and one slow-dehydrating genotype, the observed metabolic patterns cannot distinguish dehydration-associated effects from all genotype-specific effects. Additional analyses across broader germplasm panels, together with functional experiments, will therefore be required to determine the general relevance and potential roles of these candidate processes in embryo dehydration.

### 3.7. Integrated Transcriptomic and Metabolomic Analyses Identify ABA- and Ethylene-Associated Candidate Processes Linked to Contrasting Embryo Dehydration Phenotypes

**Figure 7:**
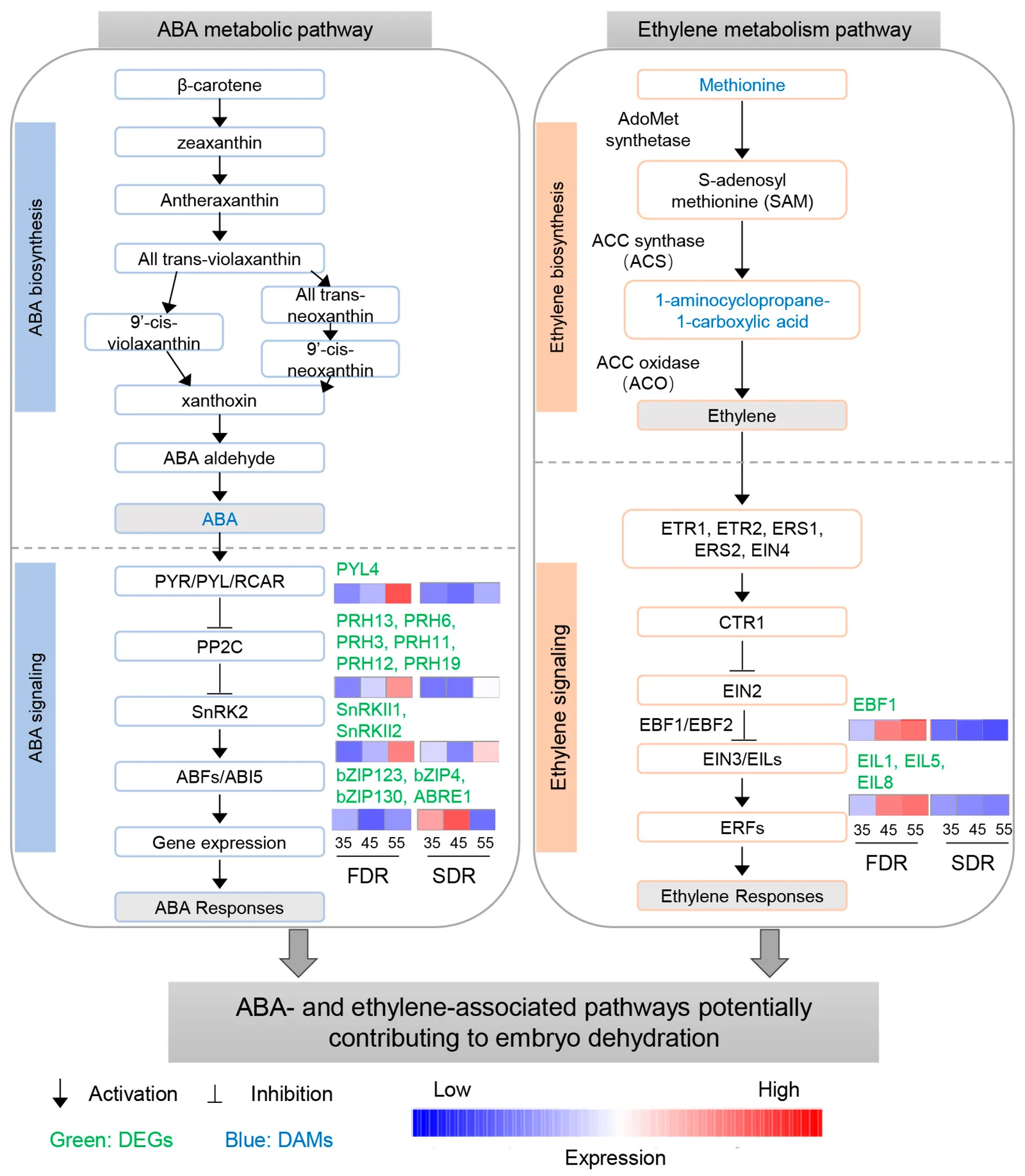
A proposed conceptual model summarizing pathway-level transcriptomic and metabolomic associations with differential embryo dehydration. The model integrates pathways in which transcriptomic and metabolomic changes converged in the present study, together with established biological relationships reported in previous studies. ABA- and ethylene-associated components and metabolic processes are presented as candidate pathways potentially involved in differential embryo dehydration. Directional relationships in the model should not be interpreted as experimentally demonstrated direct regulatory interactions or causal gene–metabolite relationships. Heatmaps depict relative expression patterns of candidate genes and relative abundance profiles of hormone-related metabolites across embryo developmental stages (35, 45, and 55 DAP). Red and blue represent relatively higher and lower expression or metabolite abundance levels, respectively. Green labels denote dehydration-associated DEGs, whereas blue labels indicate DAMs. The ABA-related section summarizes ABA-associated metabolic features and components of the PYR/PYL–PP2C–SnRK2–ABF/ABI5 signaling pathway identified in the integrated analysis. The ethylene-related section summarizes methionine-associated metabolic features and annotated components of the ETR–CTR1–EIN2–EIN3/EIL–ERF signaling pathway. Solid arrows indicate established biochemical or signaling relationships adopted from previous knowledge, whereas dashed arrows represent indirect or multistep relationships.

To integrate the transcriptomic and metabolomic datasets, we focused on pathway-level convergence between DEGs and FDR-controlled DAMs rather than pairwise gene–metabolite correlation analysis. Genes and metabolites showing significant changes were mapped independently to KEGG pathways, and pathways represented by both transcriptomic and metabolomic changes were identified as candidate biological processes associated with contrasting embryo dehydration phenotypes. No pairwise gene–metabolite correlation analysis was performed. This integrative analysis identified pathway-level convergence in several hormone- and metabolism-related pathways, including plant hormone signal transduction and pathways associated with ABA- and ethylene-related processes (Figure 7). At the pathway level, genes annotated to hormone biosynthesis or signaling and metabolites related to these pathways were both represented among the significantly changed features between the contrasting genotypes and developmental stages. These results indicate pathway-level associations between transcriptional and metabolic variation but do not establish direct regulatory or causal relationships between individual genes and metabolites.

## 4. Discussion

Kernel dehydration is a genetically regulated developmental process that influences kernel moisture at harvest, the efficiency of mechanical harvesting, and post-harvest drying requirements in maize. Previous studies have primarily focused on whole-kernel moisture dynamics and have identified numerous quantitative trait loci (QTLs) and candidate genes associated with dehydration-related traits through linkage mapping and genome-wide association studies [5,6,40,41]. However, maize kernels comprise multiple tissues with distinct developmental origins, metabolic activities, and physiological functions, and the extent to which individual tissues contribute to variation in kernel dehydration remains largely unclear.

Here, we show that embryo dehydration dynamics are closely associated with overall kernel dry-down. The similar developmental patterns observed for embryo and whole-kernel dehydration, together with the strong association between embryo and kernel dehydration rates, indicate coordinated variation between these traits. However, these relationships do not, by themselves, establish a quantitative or causal contribution of embryo water loss to whole-kernel dehydration. In addition, integrated transcriptomic and metabolomic analyses identified developmental and genotype-dependent molecular patterns associated with contrasting embryo dehydration phenotypes, including candidate metabolic and hormone-related processes. Collectively, these findings provide an embryo-focused framework for investigating tissue-specific molecular variation associated with maize kernel dehydration and for prioritizing candidate molecular processes for future functional studies.

### 4.1. Embryo Dehydration Dynamics Are Closely Associated with Maize Kernel Dry-Down Performance

Rapid kernel dehydration following physiological maturity is an important agronomic trait that influences harvest readiness, mechanical harvesting efficiency, post-harvest drying requirements, and grain storage performance. Previous genetic studies have identified multiple genomic loci associated with kernel moisture content and dehydration rate, indicating a complex genetic architecture underlying variation in kernel dry-down [7,8,41,42]. During kernel maturation, different tissues undergo coordinated yet distinct developmental programs involving metabolic transitions, stress adaptation, and acquisition of desiccation tolerance [43,44]. In addition to serving as the progenitor of the next generation, the embryo is a metabolically active tissue involved in seed maturation and environmental adaptation [45,46]. Our results show that embryo dehydration dynamics closely parallel those of whole-kernel dehydration, and embryo dehydration rate is strongly associated with kernel dehydration rate. These observations indicate coordinated variation between embryo dehydration and whole-kernel dry-down and suggest that embryo dehydration dynamics are associated with natural variation in maize dry-down performance and warrant further investigation as a tissue-specific physiological feature associated with kernel dehydration. However, the correlations observed in the present study do not by themselves establish a quantitative or causal contribution of embryo water loss to whole-kernel dehydration.

Importantly, our results indicate that kernel dehydration is accompanied by distinct yet developmentally coordinated moisture dynamics among different kernel tissues. The observed relationships among embryo, endosperm, and whole-kernel dehydration traits suggest that tissue-specific dehydration patterns may reflect broader variation in kernel dry-down (Figure 1, Figures S1 and S2). Associations between embryo dehydration traits and kernel developmental parameters further indicate that embryo moisture dynamics change during the transition from active grain filling to maturation. Together, these observations suggest that embryo moisture-related traits warrant further evaluation as potential physiological indicators associated with kernel dehydration capacity (Figure 1, Figures S1 and S2). Validation across broader germplasm panels and multiple environments will be required before their potential utility in breeding can be established.

### 4.2. Differential Embryo Dehydration Capacity Is Associated with Stage-Specific Transcriptional Reprogramming

Although numerous genetic loci associated with maize kernel dehydration have been identified through linkage mapping and genome-wide association studies, previous research has primarily focused on whole-kernel dehydration. By contrast, the embryo is a physiologically active tissue that undergoes extensive developmental and metabolic reprogramming during seed maturation [47,48]. Kernel maturation and dehydration are accompanied by substantial transcriptional changes associated with storage metabolism, stress adaptation, and developmental transitions [45,49]. However, whether variation in embryo transcriptional regulation contributes to differences in dehydration capacity remains unclear.

In this study, comparative transcriptomic analysis revealed distinct developmental- and genotype-dependent transcriptional patterns among the four maize inbred lines (Figure 2). Differences in the numbers of DEGs should not be interpreted as direct evidence of greater or lower transcriptional plasticity. Instead, the transcriptomic data indicate that the two FDR genotypes exhibited greater concordance in late-stage transcriptional responses than the two SDR genotypes. This observation suggests that specific late-stage expression profiles may be reproducibly associated with the faster-dehydrating phenotype. However, functional experiments will be required to determine whether individual genes or coordinated gene-expression patterns play causal roles in embryo dehydration. WGCNA further identified gene modules associated with embryo dehydration dynamics and enriched in multiple metabolic and signaling pathways (Figure 5 and Figure S6). The MEbrown module was predominantly enriched in pathways related to fatty acid biosynthesis, glycolysis, carbohydrate metabolism, and energy metabolism, indicating that genes involved in these metabolic pathways show coordinated expression patterns during embryo maturation and are correlated with differences in dehydration dynamics (Figure 4C and Figure S6). In contrast, the MEbrown4 module was enriched in pathways related to hormone signaling and stress-responsive pathways, highlighting hormone signaling and stress-responsive pathways as candidate signaling pathways associated with developmental- and genotype-dependent variation in embryo dehydration (Figure 4D and Figure 5).

Together, these findings indicate that contrasting embryo dehydration phenotypes are accompanied by coordinated changes in the expression of multiple genes and biological pathways. The identified co-expression modules provide a useful framework for prioritizing candidate genes and molecular processes for future functional analyses and for further investigating tissue-specific molecular variation associated with maize kernel dehydration.

### 4.3. Metabolic Processes Associated with Contrasting Embryo Dehydration Dynamics

Kernel maturation and dehydration are accompanied by substantial metabolic changes that may support cellular maintenance during progressive water loss [10,43,50]. The transcriptomic and metabolomic analyses performed in this study therefore provide an opportunity to characterize metabolic patterns associated with contrasting embryo dehydration phenotypes. Genes involved in fatty acid biosynthesis and lipid remodeling, including members of the *ACC*, *FAB*, *FAT*, and *LACS* families, exhibited distinct expression patterns between FDR and SDR embryos, indicating that lipid metabolism-related transcriptional patterns are associated with developmental- and genotype-dependent differences in embryo dehydration (Figure 5 and Figure S6). Dynamic remodeling of membrane lipids is important for maintaining membrane integrity and fluidity during cellular dehydration and may help mitigate dehydration-associated structural damage [51].

In addition to lipid metabolism, carbohydrate and energy metabolism were prominently represented among the dehydration-associated transcriptional patterns [52]. The expression patterns observed for genes involved in glycolysis, pyruvate metabolism, and the TCA cycle identify these pathways as candidate metabolic processes associated with cellular changes during progressive water loss [10]. Similar metabolic transitions have been observed during kernel maturation, where carbon metabolism shifts from growth-associated processes toward stress protection and maturation-related programs [53]. Metabolomic profiling further revealed differential accumulation of lipid-related metabolites, organic acids, phenylpropanoids, and glutathione-associated compounds between HCL645 and 911 embryos (Figure 6B and Figure S7). These differences highlight multiple metabolic categories that vary between the two representative genotypes during embryo maturation. Because the metabolomic analysis included only one FDR genotype and one SDR genotype, the observed differences should be interpreted as genotype-specific metabolic patterns associated with contrasting dehydration phenotypes rather than as universally established adaptive mechanisms of embryo dehydration.

Collectively, the transcriptomic and metabolomic analyses identified multiple metabolic categories, including lipid metabolism, organic acid, and glutathione-associated metabolism, that were associated with developmental- and genotype-dependent differences during embryo maturation. These patterns provide candidate metabolic processes for future functional and biochemical studies aimed at determining their potential relationships with embryo dehydration.

### 4.4. ABA- and Ethylene-Related Processes Are Associated with Contrasting Embryo Dehydration Dynamics

Phytohormones integrate developmental signals and environmental responses during kernel maturation and dehydration [5,45,54]. Among these hormones, ABA plays an important role in kernel maturation and desiccation tolerance through the PYR/PYL–PP2C–SnRK2 signaling cascade [34,35]. Genes annotated with ABA biosynthesis, perception, and signaling, including *NCED*, *ABA2*, *PYR/PYL*, *PP2C*, and *SnRK2* family members, exhibited differential expression patterns across developmental stages and genotypes (Figure 5 and Figure 7). The coordinated transcriptomic and metabolomic patterns observed in this study highlight ABA-associated metabolism and signaling processes as candidate processes associated with genotype- and developmental-stage-dependent variation in embryo dehydration. However, the present data do not establish a causal role for ABA signaling in determining embryo dehydration capacity.

Ethylene-associated molecular patterns were also identified through the integrated analyses. Ethylene regulates developmental transitions and stress adaptation primarily through the EIN2–EIN3/EIL signaling pathway [26]. Genes associated with ethylene biosynthesis and signaling, including members of the *ACS*, *ACO*, *EIN3/EIL*, and *EBF* families, together with metabolites related to ethylene precursor metabolism, exhibited developmental- and genotype-dependent patterns in the integrated datasets (Figure 5 and Figure 7). However, the present data do not establish whether ABA or ethylene acts upstream of the observed metabolic changes, nor do they define a causal regulatory hierarchy linking hormone signaling, transcriptional changes, and metabolic processes. Therefore, ABA- and ethylene-related pathways are considered candidate regulatory processes that warrant further functional investigation. These observations provide an embryo-focused framework for prioritizing ABA- and ethylene-associated candidate processes for future studies of the molecular basis of maize kernel dehydration.

## 5. Conclusions

Collectively, this study characterized developmental dehydration dynamics in maize kernels, endosperms, and embryos and revealed close physiological associations between embryo and whole-kernel dehydration during kernel maturation. Two-way ANOVA showed that genotype, developmental stage, and their interaction significantly affected moisture content and dehydration rate across tissues. Comparative transcriptomic and WGCNA analyses identified developmental- and genotype-dependent gene-expression patterns associated with contrasting embryo dehydration phenotypes, including processes related to fatty acid metabolism, carbohydrate metabolism, MAPK signaling, and plant hormone signaling. FDR-controlled metabolomic analysis of two representative contrasting genotypes, HCL645 and 911, further revealed genotype- and developmental-stage-dependent differences in metabolite profiles, particularly those associated with organic acid metabolism, amino acid metabolism, transport-associated processes, and secondary metabolism. Pathway-level integration of the transcriptomic and metabolomic datasets, rather than pairwise gene–metabolite correlation analysis, highlighted ABA- and ethylene-associated candidate processes associated with variation in embryo dehydration. Together, these results provide an embryo-focused framework for characterizing physiological associations and prioritizing candidate genes and metabolic processes associated with variation in embryo dehydration. However, direct experimental validation, together with evaluation across broader germplasm panels and environmental conditions, will be required to determine the causal roles, general relevance, and potential breeding value of the identified candidate factors.

## Data Availability

All the data from this study are included in the article and Supplementary Materials. For further inquiries, please contact the corresponding author.

## Supplementary Materials

The following supporting information can be downloaded at https://www.mdpi.com/article/10.3390/genes17101196/s1: Figure S1: Developmental changes in moisture content and dehydration rate of whole kernels and endosperms in four maize inbred lines with contrasting dehydration phenotypes. Figure S2: Pearson correlation matrix of dehydration-related and agronomic traits across maize kernels, endosperms, and embryos. Figure S3: Quality assessment and reproducibility of the embryo transcriptome dataset. Figure S4: Volcano plots of DEGs in maize embryos during the 45 vs. 35 DAP developmental transition. Figure S5: Volcano plots of DEGs in maize embryos during the 55 vs. 45 DAP developmental transition. Figure S6: Expression patterns of representative genes associated with fatty acid biosynthesis and glycolysis/gluconeogenesis in the dehydration-associated MEbrown module. Figure S7: PLS-DA model validation for metabolomic profiles of HCL645 and 911 embryos. Figure S8: Pathway enrichment analysis of DAMs identified in HCL645 and 911 embryos. Table S1A: Agronomic traits and moisture-related physiological parameters of four maize inbred lines with contrasting dehydration phenotypes. Table S1B: Two-way ANOVA and one-way ANOVA results for moisture-related physiological traits in kernels, endosperms, and embryos of four maize inbred lines with contrasting dehydration phenotypes. Table S2: DEGs identified in maize embryos during the 45 vs. 35 DAP developmental transition in four maize inbred lines with contrasting dehydration phenotypes. Table S3: DEGs identified in maize embryos during the 55 vs. 45 DAP developmental transition in HCL645 with contrasting dehydration phenotypes. Table S4: Candidate dehydration-associated DEGs involved in fatty acid biosynthesis and glycolysis/gluconeogenesis in maize embryos. Table S5: Candidate dehydration-associated DEGs involved in MAPK signaling and plant hormone signal transduction in maize embryos. Table S6: Quantitative metabolite profiles and annotations identified in HCL645 and 911 maize embryos during kernel maturation. Table S7: DAMs identified in HCL645 embryos during the 45 vs. 35 DAP developmental transition. Table S8: DAMs identified in 911 embryos during the 55 vs. 45 DAP developmental transition.

## Abbreviations

The following abbreviations are used in this manuscript:

DAP	Days after pollination
WGCNA	Weighted gene co-expression network analysis
KMC	Kernel moisture content
KDR	Kernel dehydration rate
LEA	Late embryogenesis abundant
GWAS	Genome-wide association study
QTL	Quantitative trait locus
QTNs	Quantitative trait nucleotides
SDR	Slow-dehydrating rate
FDR	Fast-dehydrating rate
MC	Moisture content
DR	Dehydration rate
EmDR	Dehydration rates of embryo
EnDR	Dehydration rates of endosperm
EnMC	Endosperm moisture content
EmMC	Embryo moisture content
100 KW	100-kernel weight
KWE	Kernel weight per ear
KW	Kernel width
KT	Kernel thickness
AUDDC	Area under the dry-down curve
DEG	Differentially expressed gene
TCA cycle	Tricarboxylic acid cycle
PFK1	Phosphofructokinase
PYK	Pyruvate kinase
HEX	Hexokinase
ENO1	Enolase
PGK	Phosphoglycerate kinase
TPI	Triosephosphate isomerase
ALD1	Fructose-bisphosphate aldolase
ADH	Alcohol dehydrogenase
PDC	Pyruvate decarboxylase
SnRKII	SNF1-related protein kinase 2
EIL	Ethylene-insensitive-like
EBF	EIN-binding F-box protein
DAM	Differentially accumulated metabolites
PCA	Principal component analysis
ACO	ACC oxidase
SAM	S-adenosylmethionine
ACS	ACC synthase
ACC	1-aminocyclopropane-1-carboxylic acid
