# Supplementary material for: Integrative Transcriptomic and Metabolomic Analyses Reveal Molecular Processes Associated with Differential Embryo Dehydration in Maize
Source: Genes (Basel). 2026 Sep 27;17(10):1196. doi: 10.3390/genes17101196 (PMC13704109; doi:10.3390/genes17101196)
Supplement: Supplementary file 1 [file genes-17-01196-s001.zip › genes-4541026-Supplementary Figures.pdf]

Supplementary Figures

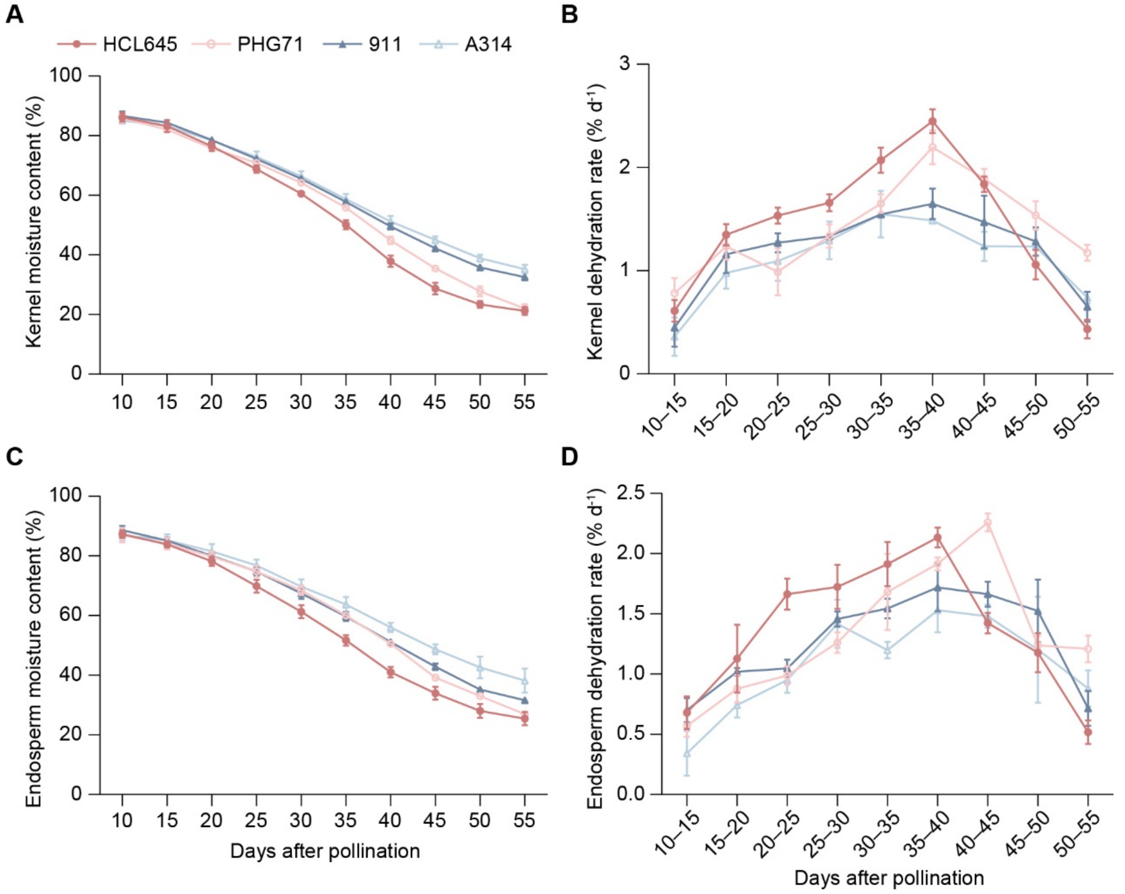

Figure S1. Developmental changes in moisture content and dehydration rate of whole kernels and endosperms in four maize inbred lines with contrasting dehydration phenotypes. (A) Whole-kernel moisture content from 10 to 55 DAP in HCL645, PHG71, 911, and A314. (B) Whole-kernel dehydration rate during successive developmental intervals. (C) Endosperm moisture content from 10 to 55 DAP. (D) Endosperm dehydration rate during successive developmental intervals. HCL645 and PHG71 represent the FDR group, whereas 911 and A314 represent the SDR group. Data are presented as means ± SD (n = 3 independent biological replicates). Genotype, developmental stage, and their interaction were evaluated by two-way ANOVA, followed by Tukey's HSD test where appropriate.

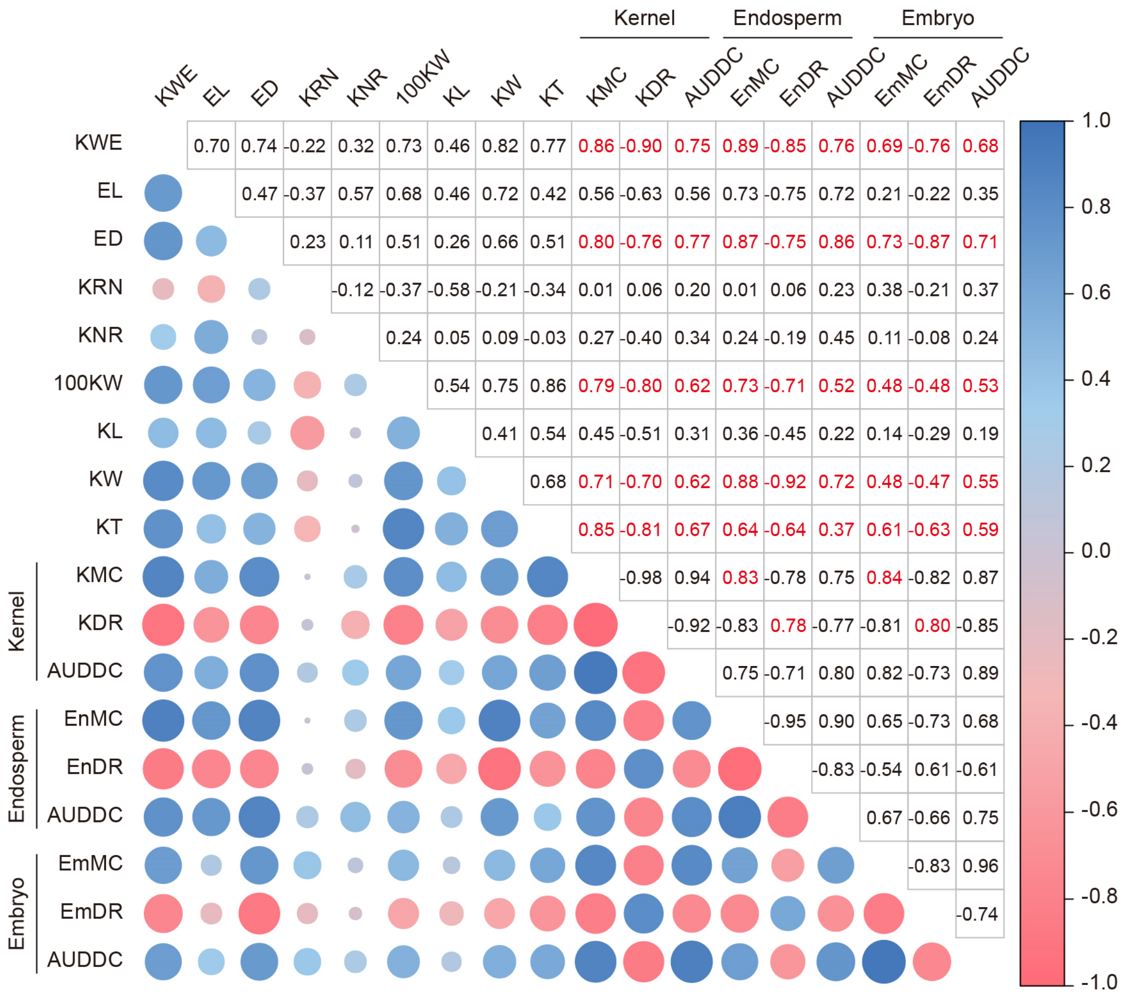

Figure S2. Pearson correlation matrix of dehydration-related and agronomic traits across maize kernels, endosperms, and embryos. Pairwise Pearson correlation coefficients were calculated among kernel- and ear-related traits (KWE, EL, ED, KRN, KNR, 100KW, KL, KW, and KT) and moisture-related traits of kernels (KMC, KDR, and AUDDC), endosperms (EnMC, EnDR, and AUDDC), and embryos (EmMC, EmDR, and AUDDC). The upper triangle presents numerical correlation coefficients, whereas the lower triangle represents correlation strength and direction using colored circles. Positive and negative correlations are indicated by blue and red, respectively. The color gradient ranges from −1.0 to 1.0.

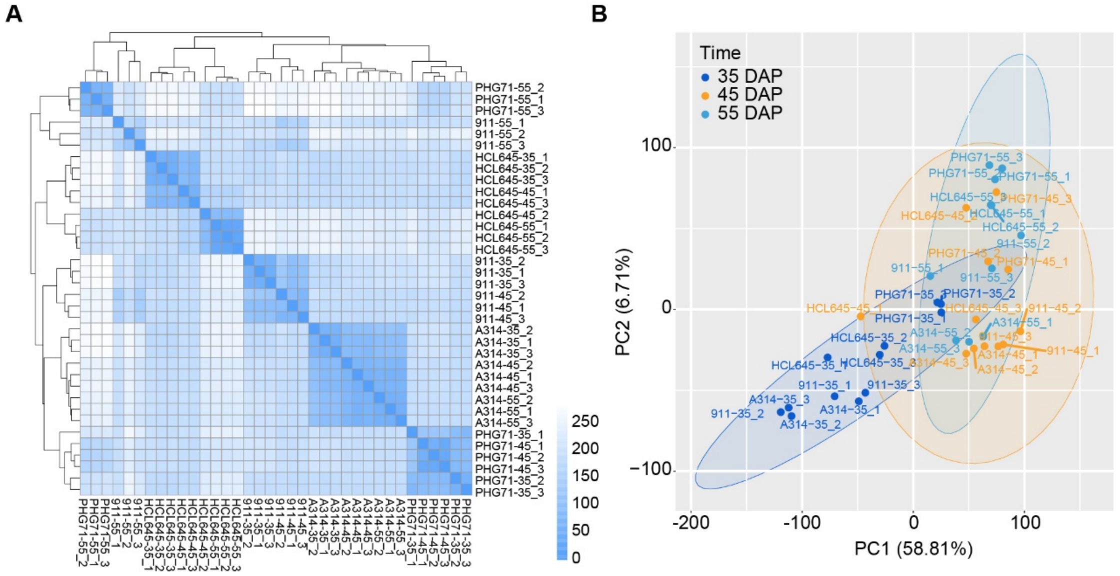

Figure S3. Quality assessment and reproducibility of the embryo transcriptome dataset. (A) Pearson correlation heatmap showing pairwise correlations among the 36 RNA-seq libraries generated from four maize inbred lines at three developmental stages, with three independent biological replicates for each genotype × developmental-stage combination. (B) Principal component analysis (PCA) of embryo transcriptome profiles across three developmental stages (35, 45, and 55 DAP). PC1 and PC2 explain 58.81% and 6.71% of the total variance, respectively. Biological replicates from the same genotype and developmental stage generally showed similar expression profiles. Samples are displayed with 95% confidence ellipses.

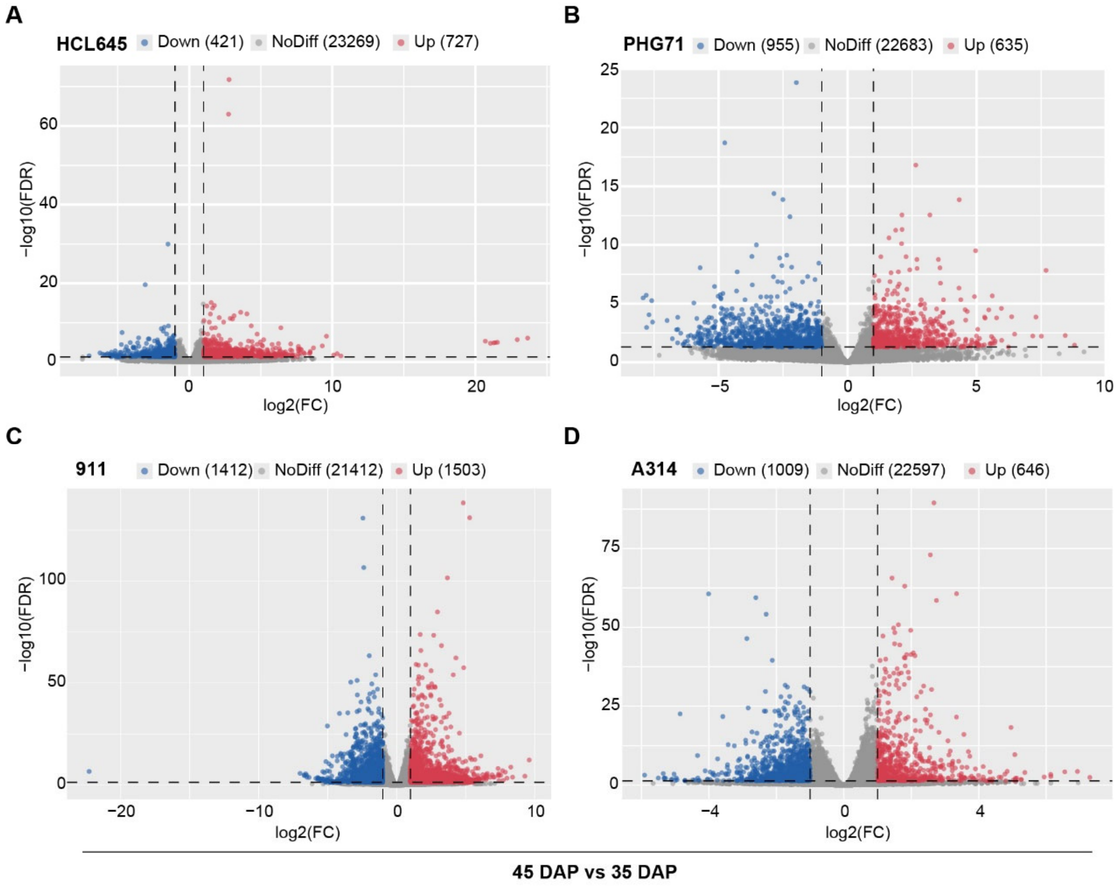

Figure S4. Volcano plots of DEGs in maize embryos during the 45 vs. 35 DAP developmental transition. (A–D) Volcano plots showing DEGs identified between 45 and 35 DAP in embryos of HCL645 (A), PHG71 (B), 911 (C), and A314 (D). The x-axis represents log2(fold change) (log2FC), and the y-axis represents statistical significance expressed as −log10(FDR). Red dots indicate significantly upregulated genes, blue dots indicate significantly downregulated genes, and gray dots represent genes without significant differential expression. Vertical dashed lines indicate the thresholds of |log2FC| = 1, and the horizontal dashed line indicates the FDR threshold. The numbers of downregulated, nonsignificant, and upregulated genes are shown in the upper-left corner of each plot.

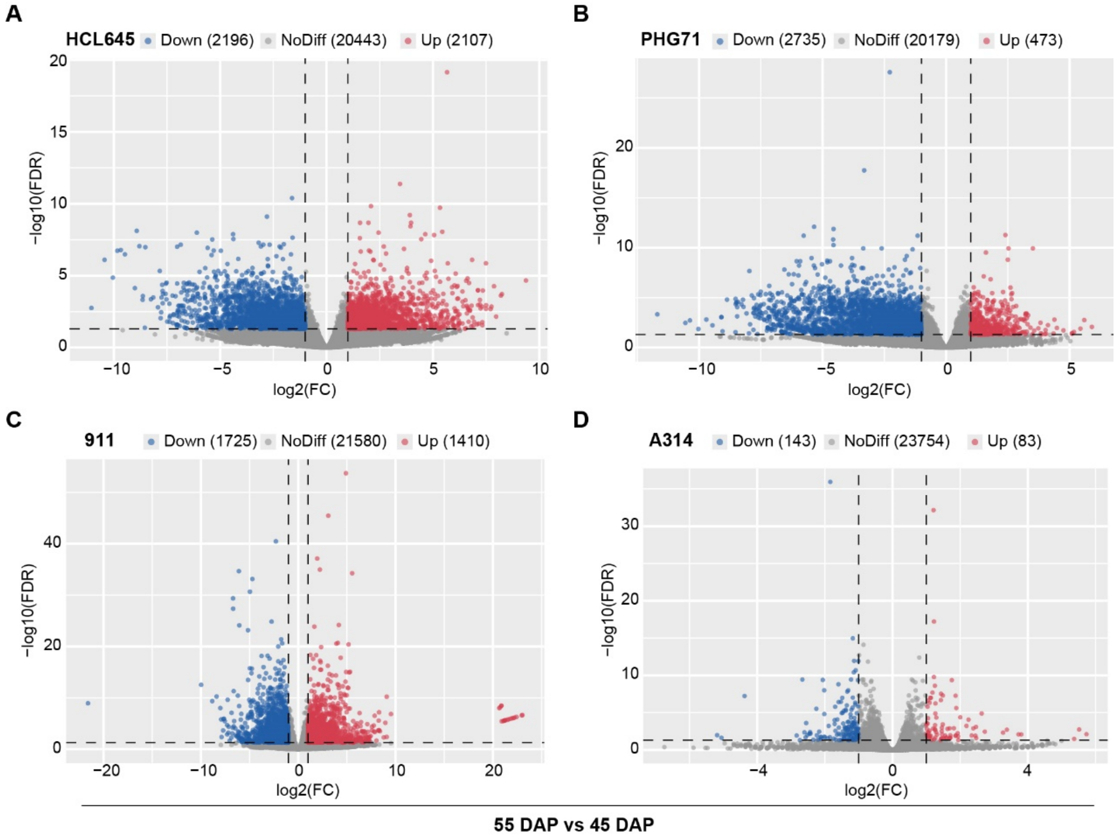

Figure S5. Volcano plots of DEGs in maize embryos during the 55 vs. 45 DAP developmental transition. (A–D) Volcano plots showing DEGs identified between 55 and 45 DAP in embryos of HCL645 (A), PHG71 (B), 911 (C), and A314 (D). The x-axis represents log2(fold change) (log2FC), and the y-axis represents statistical significance expressed as −log10(FDR). Red dots indicate significantly upregulated genes, blue dots indicate significantly downregulated genes, and gray dots represent genes without significant differential expression. Vertical and horizontal dashed lines indicate the thresholds of |log2FC| = 1 and FDR, respectively. The numbers of downregulated, nonsignificant, and upregulated genes are shown in the upper-left corner of each plot.

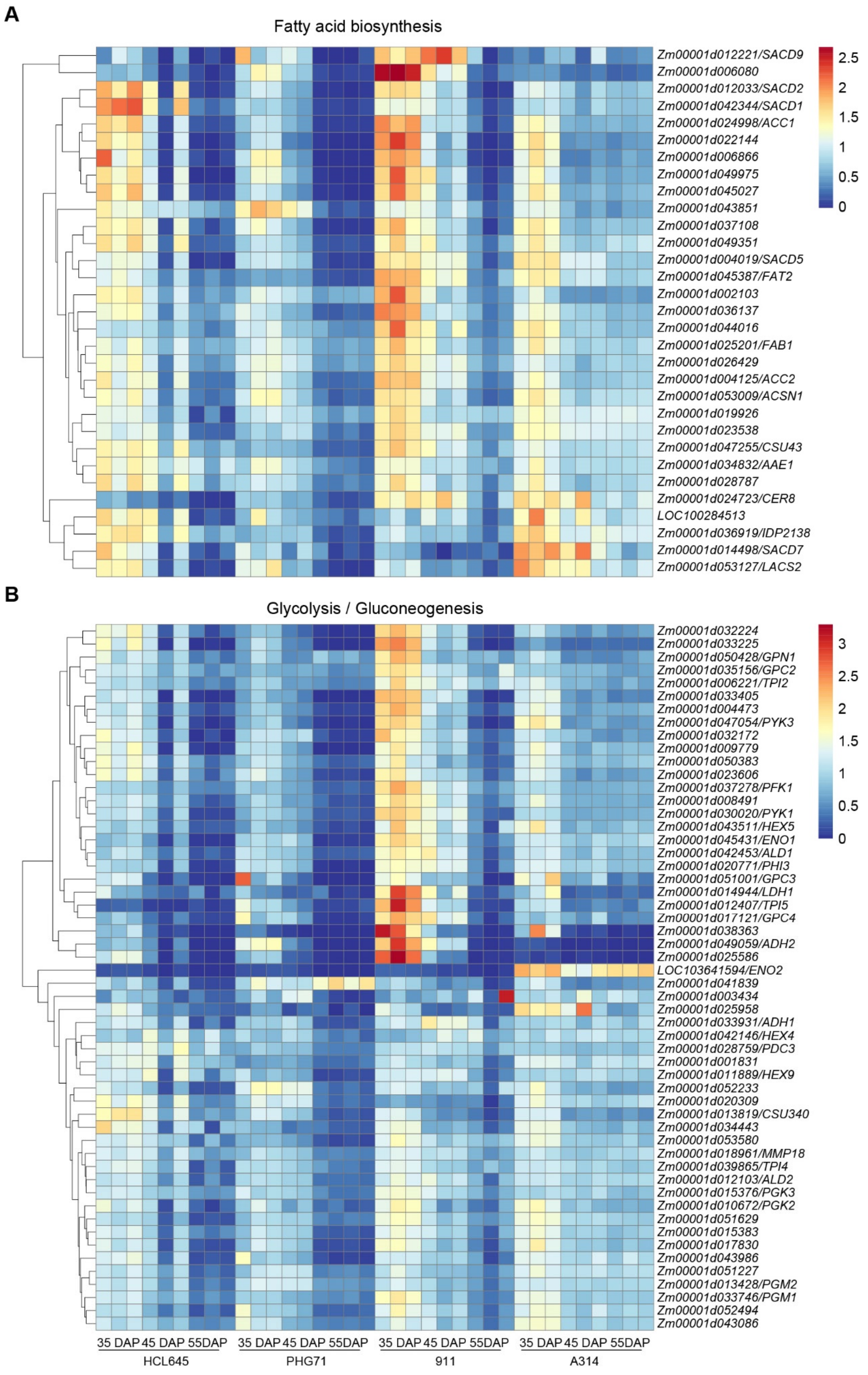

Figure S6. Expression patterns of representative genes associated with fatty

acid biosynthesis and glycolysis/gluconeogenesis in the dehydration-associated MEbrown module. (A) Heatmap showing the expression patterns of DEGs enriched in the fatty acid biosynthesis pathway within the MEbrown module. (B) Heatmap showing the expression patterns of DEGs enriched in the glycolysis / gluconeogenesis pathway within the MEbrown module. Rows represent individual DEGs, and columns represent biological replicates of HCL645, PHG71, 911, and A314 embryos at 35, 45 and 55 DAP. The color scale represents relative expression levels, with red indicating higher expression and blue indicating lower expression.

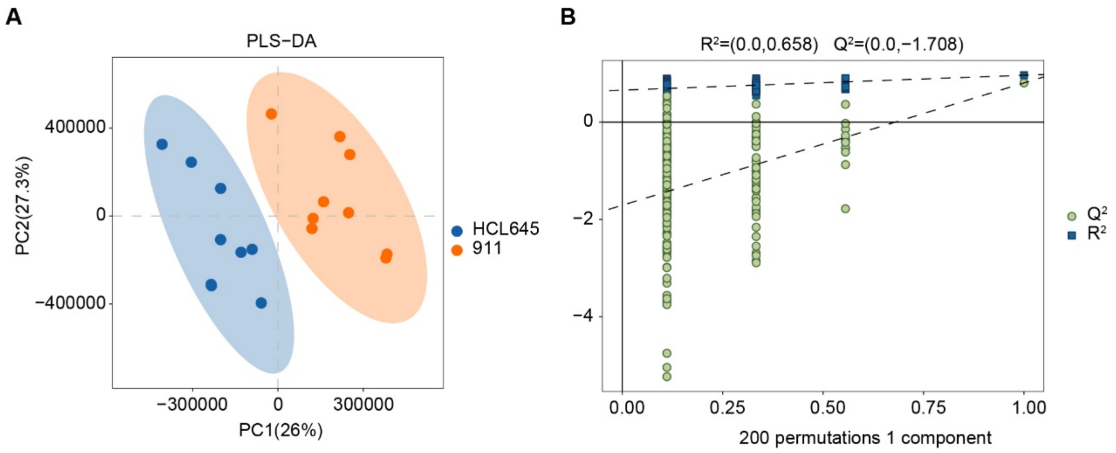

Figure S7. PLS-DA model validation for metabolomic profiles of HCL645 and 911 embryos. (A) PLS-DA score plot showing separation between HCL645 and 911 embryos based on Pareto-scaled metabolomic data. The number of latent components was selected by 7-fold cross-validation. (B) Permutation test plot (200 permutations) for the PLS-DA model. The model showed $R^2X(cum) = 0.585$, $R^2Y(cum) = 0.964$, and $Q^2(cum) = 0.806$. The permutation test yielded an R^2 intercept of 0.658 and a Q^2 intercept of −1.708 ($P < 0.05$), indicating no obvious overfitting.

A

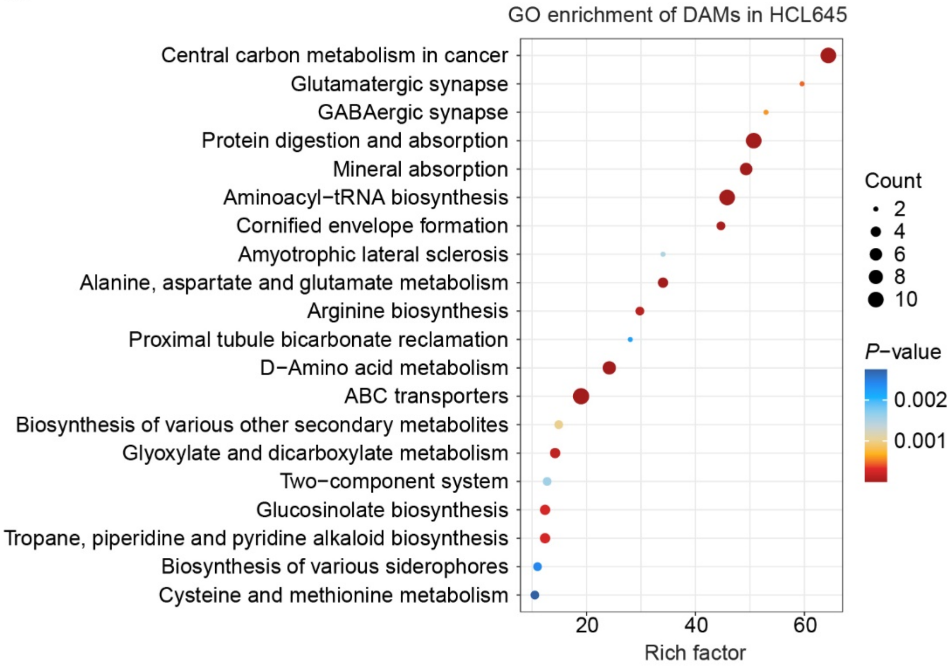

B

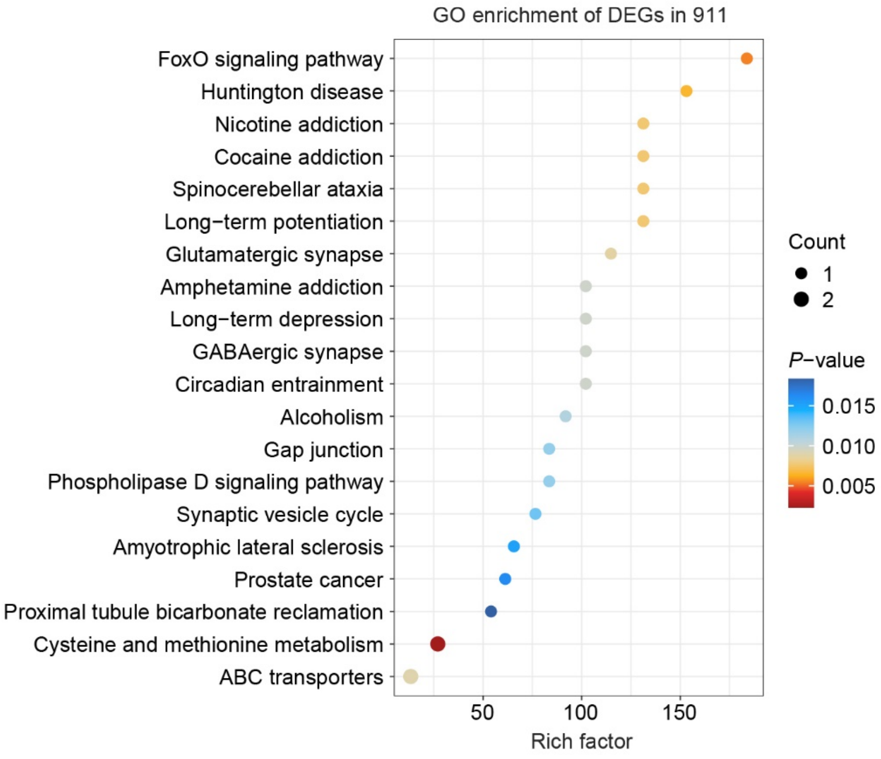

Figure S8. Pathway enrichment analysis of DAMs identified in HCL645 and 911 embryos. (A) GO enrichment bubble plot of DAMs identified in HCL645 embryos. (B) GO enrichment bubble plot of DAMs identified in 911 embryos. The vertical axis represents enriched GO terms, and the horizontal axis represents the rich factor. Bubble size indicates the number of DAMs associated with each GO term, and bubble color represents the statistical significance of the enrichment based on the *P*-value.
